# Genotype–phenotype correlations with autism spectrum disorder-related traits in Noonan syndrome and Noonan syndrome with multiple lentigines: a cross-sectional study

**DOI:** 10.1186/s13229-025-00681-1

**Published:** 2025-10-11

**Authors:** Chloe Alexa McGhee, Julia R. Plank, Luca Pannone, Odeya Russo, Naomi Fuhrmann, Aurora Ruggeri, Francesca Clementina Radio, Simone Martinelli, Marco Tartaglia, Tamar Green

**Affiliations:** 1 Department of Psychiatry & Behavioral Sciences, Stanford University, Stanford, USA; 2 Molecular Genetics and Functional Genomics Research Division, Bambino Gesù Children’s Hospital, IRCCS, Rome, Italy; 3 Department of Oncology and Molecular Medicine, Istituto Superiore Di Sanità, Rome, Italy

**Keywords:** Noonan syndrome, Noonan syndrome with multiple lentigines, RASopathies, Genotype-phenotype correlations, Behavior, PTPN11

## Abstract

**Background:**

Noonan syndrome (NS) and Noonan syndrome with multiple lentigines (NSML) are neurodevelopmental conditions caused by genetic variants leading to upregulated signaling in the RAS-MAPK pathway. While previous research has focused on genetic variability in cognitive and cardiac phenotypes, behavioral phenotypes, and their correlations across genetic variants and within the *PTPN11* gene remain poorly characterized.

**Methods:**

This study included 121 individuals with NS (*PTPN11*: 88, *SOS1*: 18, *RAF1*: 6, *KRAS*: 2, *RIT1*: 3, *NRAS*: 2, *LZTR1*: 2, *SOS2*: 1) and seven individuals with NSML (*PTPN11*), compared to age- and sex-matched typically developing (TD) (N = 71). Behavioral questionnaires assessed social responsiveness and ASD-related traits (using SRS-2), and emotional problems (using CBCL) to identify genetic variant-specific behavioral profiles. Biochemical profiling of SHP2 activity in *PTPN11*-associated NS variants examined genotype–phenotype relationships.

**Results:**

Compared to TD individuals, those with *PTPN11*-associated NS, NSML, and *SOS1*-associated NS exhibited clinically elevated scores, indicating increased ASD-related behaviors, poorer social functioning, and heightened emotional problems. Genetic variant comparisons revealed that individuals with *PTPN11*-associated NS and NSML exhibited greater ASD-related challenges than those with *RAF1*. Individuals with NSML exhibit elevated attention problems compared to all other genetic groups. Logistic regression results suggested each one-unit increase in SHP2 fold activation for *PTPN11*-associated NS corresponded to a 64% higher likelihood of markedly elevated restricted and repetitive behaviors, suggesting genotype–phenotype links.

**Limitations:**

Small sample sizes for rarer variants, leading to unequal group sizes across subgroups, with *PTPN11* variants comprising most of the NS group. Future research should address these sampling constraints and conduct functional studies to clarify variant impacts. Longitudinal assessments could elucidate behavioral phenotype trajectories.

**Conclusions:**

This study underscores the importance of genetic variant-specific research to understand unique behavioral phenotypes in NS and NSML. Our findings indicate a higher risk for ASD-related symptoms in *PTPN11*-associated NS and NSML compared to other variants. Additionally, individuals with *PTPN11*-associated NS and higher SHP2 fold activation exhibited greater impairments in restricted and repetitive behaviors, suggesting SHP2 activation variations may contribute to phenotypic variability. By linking ASD-related symptoms to biochemical predictors in *PTPN11*-associated NS, this study may inform future targeted treatment approaches.

**Supplementary Information:**

The online version contains supplementary material available at 10.1186/s13229-025-00681-1.

## Background

RASopathies are a group of clinically related disorders caused by germline variants in the RAS-mitogen-activated protein kinase (RAS-MAPK) signaling pathway [1, 2]. This disease family includes an increasing number of conditions, among which Noonan syndrome (NS, MIM PS163950) is the most common (estimated prevalence of 1:2000) and clinically variable. NS is caused most often by missense variants in *PTPN11* (~ 50%), *SOS1* (~ 10%), and *LZTR1* (~ 10%), while variants in *RAF1, KRAS, RIT1, MRAS*, *RRAS2*, *SPRED2*, *BRAF*, *MAP2K1*, *NRAS,* and *SOS2* account for an additional 10–20% of cases, leading to an enhanced signaling through the RAS-MAPK pathway [3–5]. Noonan syndrome with multiple lentigines (NSML, MIM 151100) is clinically related and allelic to NS and is caused by a different class of missense variants in *PTPN11* [6], which cause impaired catalytic activity of the encoded protein, SHP2, and a more articulated dysregulation of intracellular signaling, including the upregulation of the phosphoinositide 3-kinase (PI3K)-AKT-mTOR cascade [7, 8].

Children with NS and NSML experience a range of cardiac and physical abnormalities, as well as cognitive and social impairments; up to 30% present with ASD-related behavior [9–12]. Previous research has established associations between specific NS-causing genes/variants and distinct cardiac, physical, and cognitive phenotypes [13–16]. For instance, NS-associated *PTPN11* variants have been linked to pulmonary valve stenosis [15, 17], while NSML*-*associated *PTPN11* variants and those of *RAF1* implicated in NS are commonly associated with hypertrophic cardiomyopathy [16]. Within children with NS and NSML [11, 18–22], most demonstrate intellectual functioning within the average range, though there is some variation depending on genotype. For example, individuals with *RAF1* variants show borderline to average IQ scores, while those with *SOS1* variants typically exhibit average IQ scores and fewer cognitive delays [11, 17, 21, 22]. Individuals with *KRAS* variants often experience considerable cognitive delays [23–25]. While these findings testify to the occurrence of genotype–phenotype correlations in the context of cognitive and cardiac profiles, investigations into the behavioral manifestations associated with NS and NSML-related gene variants remain poorly defined.

Behavioral challenges are prevalent in NS, with approximately 48% of youth exhibiting attention deficit hyperactivity disorder (ADHD), and frequent impairments in attention, executive functioning, emotional regulation, and social behaviors [13, 14, 21, 26, 27]. The prevalence of autism spectrum disorder (ASD) in NS is estimated to range from 12 to 30% [14, 28–30], and elevated ASD symptoms have been observed in 25% of children with NSML [18]. Variant-specific differences have also emerged; for example, individuals with *SOS1* variants generally score below the clinical threshold for autistic behaviors [12, 20], whereas those with NS-associated *PTPN11* variants tend to exhibit subthreshold autism symptoms alongside more pronounced impairments in emotional regulation [21, 31]. Despite these findings, most prior studies aggregated all NS-causing genes to address small sample sizes, thereby overlooking critical genetic variant-specific behavioral differences. Previous behavioral and cognitive comparisons have primarily focused on *PTPN11*- and *SOS1*-associated NS variants, with sample sizes ranging from 6 to 66 for *PTPN11* and 1 to 17 for *SOS1*. In contrast, rarer NS variants—such as *RAF1*, *KRAS*, *RIT1*, *LZTR1*, *NRAS*, *SOS2*, and *PTPN11*-associated NSML—remain largely understudied. Typical sample sizes for these variants range from 2 to 7 for *RAF1*, 2 to 6 for *PTPN11*-associated NSML, and 1 to 4 for other rare mutations. Studies focusing on cognitive and behavioral phenotypes in NS and NSML often combine these rarer variants, which limits the ability to capture behavioral heterogeneity—an issue our study directly addresses.

Some genotype–phenotype relationships have also been identified at the variant level for cognitive and cardiac phenotypes in NS; however, few studies have comprehensively investigated the differential behavioral profiles associated with the unique functional consequences of individual variants, particularly in the case of *PTPN11*. Its encoded protein, SHP2, is a non-receptor-type tyrosine phosphatase characterized by two tandemly arranged SH2 domains and a single catalytic domain [32]. Most NS-associated *PTPN11* variants cluster within regions of the protein that regulate the switch between its inactive and active conformations. These variants upregulate SHP2 function by favoring its active conformation and leading to a gain-of-function effect on the RAS-MAPK pathway. Importantly, these variants differ in their impact on SHP2, with some causing modest increases in activation and others more substantial activation, suggesting that such a differential effect, suggesting that such a differential impact may contribute to explaining the observed variability in cognitive, cardiac, and behavioral phenotypes [33, 34]. This phenotypic heterogeneity may stem from differential effects on signaling [35] resulting from quantitative and qualitative variations in SHP2 activation (*e.g.*, basal and stimulated activity and their ratio classified as fold activation) [36, 37]. For example, investigations into common *PTPN11* pathogenic variants such as N308D and N308S indicate that individuals with these variants typically have average IQs and relatively mild cognitive delays [11, 15, 22, 38, 39]. Further studies, however, are needed to investigate the occurrence of correlations between phenotypic heterogeneity and variant-specific SHP2 functional dysregulation.

Here, we investigated the relationships between genotypes (at the gene and variant levels) and behavioral manifestations. Leveraging a large dataset of children with NS (N = 121) and NSML (N = 7), we compared behavioral profiles across a range of genetic variants compared to age- and sex-matched typically developing (TD) individuals (N = 71). By systematically characterizing the biochemical profile of the *PTPN11* variants identified in the NS group, we explored the occurrence of genotype–phenotype correlations related to the observed behavioral heterogeneity. By integrating these genetic, behavioral, and biochemical insights, our study advances the understanding of the biological mechanisms underlying phenotypic heterogeneity in NS and NSML.

## Methods

### Participants

With approval from the Institutional Review Board of the Stanford University School of Medicine, we performed a prospective study of children aged 4 to 17 years with NS and NSML (N = 128, mean age = 10.3, females = 70), and TD individuals (N = 71, mean age = 9.5, females = 39) (Table 1). All participants had molecularly confirmed clinical diagnoses.

**Table 1:** Participant Demographic Information

	NS/NSML (N = 128)	TD (N = 71)	NS/NSML vs.TD (*p*-value)
*Sex*			
Female	70 (54.7%)	39 (54.9%)	0.974
Male	58 (45.3%)	32 (45.1%)	
*Age (years)*			
Mean (SD)	10.3 (3.3)	9.5 (2.4)	0.092
Median [min, max]	10.1 [6, 17.91]	9.4 [6.03, 16.75]	
*FSIQ-2 scale*			
Mean (SD)	98.5 (13.4)	113.6 (13.6)	**6.45e**^**−09**^
Median [min, max]	100 [62, 133]	113 [85, 147]	
*VIQ*			
Mean (SD)	98.6 (11.9)	113.8 (12.5)	**6.31e**^**−15**^
Median [min, max]	99 [67, 132]	113 [85, 160]	
*PIQ*			
Mean (SD)	94.5 (13.8)	111.3 (14.7)	**1.30e**^**−12**^
Median [min, max]	96 [61, 135]	113 [70, 156]	

NS, Noonan syndrome; NSML, Noonan syndrome with multiple lentigines; TD, typically developing individuals; FSIQ, Full Scale IQ 2-scale [NS/NSML: N = 108; TD: N = 41]; VIQ, Verbal IQ [NS/NSML: N = 126; TD: N = 71]; PIQ, Performance IQ [NS/NSML: N = 100; TD: N = 71]. Bolded values represent significant *p*-values < .05. P-values are from chi-square (sex) and independent samples t-tests (age, FSIQ, VIQ, PIQ)

Children with NS and NSML were diagnosed based on variants in the following genes: *PTPN11* (N = 88 for NS; N = 7 for NSML), *SOS1* (N = 18), *RAF1* (N = 6), *RIT1* (N = 3), *KRAS* (N = 2), *NRAS* (N = 2), *LZTR1* (N = 1), and *SOS2* (N = 1) (Table 2). Specifically, for *PTPN11*-associated NS, 14 cases were inherited, 22 were de novo, and 52 had an unknown status. In *SOS1*, three cases were inherited, five were de novo, and 10 had an unknown status. For *PTPN11*-associated NSML, one case was inherited, two were de novo, and four had an unknown status. In RAF1, two cases were inherited, and four had an unknown status. For *RIT1*, one was de novo, and two had unknown status. In *KRAS*, both cases had an unknown status. For *NRAS*, one case was inherited, and one had an unknown status. In both *LZTR1* and *SOS2*, the single cases identified had unknown status.

**Table 2:** Descriptive Information for TD Individuals and Genetic Variants Causing NS and NSML

	TD (N = 71)	*PTPN11*-NS (N = 88)	*SOS1-*NS (N = 18)	*PTPN11-*NSML (N = 7)	*RAF1-*NS (N = 6)	*RIT1-*NS (N = 3)	*KRAS*-NS (N = 2)	*NRAS-*NS (N = 2)	*LZTR1-*NS (N = 2)	*SOS2-*NS (N = 1)
*Sex*										
Female	39(55%)	46(52%)	12(67%)	6(86%)	1(17%)	3(100%)	1(50%)	-	-	1(100%)
Male	32(45%)	42(48%)	6(33%)	1(14%)	5(83%)	-	1(50%)	2(100%)	1(100%)	-
*Age (years)*										
Mean[range]	9.5[6.0,16.8]	10.3[6.0–17.9]	9.3[6–16.3]	10.3[6.0–16.7]	12.6[9.0–17.4]	11.6[9.2–15.0]	10.6[7.2–14.1]	7.3[6.0–8.7]	6.5	10.9
*FSIQ-2 scale*										
Mean[range]	113.6[85–147]	96.4[62–123]	106[72–125]	101.1[86–116]	101.8[78–133]	103.7[96–109]	92.5[85–100]	112[94–130]	80	106
*VIQ*										
Mean[range]	113.8[85–160]	97.0[67–122]	102.9[85–121]	100.6[94–113]	99.8[76–132]	102[99–105]	101.5[101–102]	118[107–129]	76	104
*PIQ*										
Mean[range]	111.3[70–156]	91.2[61–111]	100.5[77–119]	100.2[92–118]	103.7[83–135]	109.3[102–122]	77.5[70–85]	106.0[83–129]	72	97
*SRS-2 Measures*										
Social responsiveness total	48.8[36.5–75]	61.7[42–93.5]	58.1[44–81]	66[57–72]	51.4[38–82.5]	62.3[57–67.5]	67.2[58–76.5]	63.8[61.5–66]	61.0	51.5
Restricted and repetitive behaviors	48.2[40–85]	62.7[43–97.5]	59.4[41–82]	65.4[56–77]	52.2[41–79.5]	60.7[53–71]	66.0[56–76]	58.5[58, 59]	64.5	50.0
Social cognition	47.8[38–69]	59.9[40–98.5]	56.5[41–77]	63.9[57–70]	51.3[40–82.5]	57.7[52–67]	62.5[59–66]	62.2[60–64.5]	55.0	52.0
Social Communication	48.9[37.5–75]	60.8[42.5–96.5]	56.9[37–81]	64.4[56–75]	51.6[38–81.5]	61.3[59.5–65]	67.8[57.5–78]	65.5[63.5–67.5]	63.0	53.0
Social motivation	50.3[37–84.5]	57.1[39–93]	54.3[41.5–75.5]	63[52–75.5]	50.7[40–66.5]	61.0[52.5–73.5]	67.8[55.5–80]	62.8[61–64.5]	55.0	52.5
*CBCL Measures*										
Anxious/depressed	55.1[50–70]	58.1[50–82]	56.7[50–78]	64.9[55.5–72]	56.7[50–77]	55.2[54.5–55.5]	59.5[50–69]	58.5[55–62]	53.0	50.5
Withdrawn/depressed	55.2[50–76]	57.5[50–83.5]	54.9[50–73.5]	58.6[51–70]	55[53–62]	59.2[51–71.5]	61.0[54–68]	63.5[62–65]	50.0	58.0
Somatic complaints	53.8[50–70]	58.5[50–79.5]	59.6[51.5–72]	67.4[50–77]	57.2[50–62.5]	61.0[55.5–67]	55.0[53–57]	60.5[59–62]	50.0	69.0
Thought problems	55.7[50–71]	61.9[50–87]	61[50–71.5]	67.4[58–72.5]	57.8[50–74]	62.5[62.5–62.5]	60.5[59.5–61.5]	60.8[59.5–62]	54.0	52.5
Attention problems	55.6[50–76.5]	62.3[50–90]	59.1[50–74]	68.7[59.5–76.5]	59.4[51–69]	63.7[56–72.5]	56.2[56–56.5]	57.0[57]	64.0	65.0
Rule-breaking behavior	54.7[50–69]	56.5[50–76]	54.8[50–68]	54.3[51–60]	54.2[50–59.5]	55.0[52.5–59]	50.5[50.5–50.5]	56.0[52–60]	50.5	51.0
Aggressive behavior	53[33–73.5]	57.6[39.5–90.5]	56.2[46.5–73]	61.1[50–70.5]	55.8[50–69]	58.5[55.5–61]	50.8[50.5–51]	60.0[56–64]	51.5	58.5

NS, Noonan syndrome; NSML, Noonan syndrome with multiple lentigines; TD, typically developing individuals; FSIQ-2 Scale, Full-Scale IQ 2-scale; VIQ, Verbal IQ; PIQ, Performance IQ; SRS-2, Social Responsiveness Scale Second Edition; CBCL, Child Behavior Checklist

Higher scores for the SRS-2 and CBCL indicate clinically elevated symptoms and greater impairment. The results report the mean and range of scores for genetic variants with more than two individuals. Genetic variants with only one individual have the reported score for each measure

All participants were recruited over six years (2018–2024) through the Noonan Syndrome Foundation and Stanford University School of Medicine’s website, parent social media networks, and advertisements. TD participants were selected to match the NS group in age and sex.

Individuals with NS or NSML and TD participants were recruited through two NIH-funded studies (#HD090209 K23 and #HD108684 R01). Children with NS, NSML, and TD were excluded if they met any of the following criteria: known diagnosis of a major psychiatric disorder (e.g., psychotic disorders, major mood disorders, or previously documented full-scale IQ < 70), a current neurological disorder (e.g., seizures, history of head trauma, a diagnosis of gross structural brain malformations, or any known tumors or cerebellar, brainstem, tectal plate, and basal ganglia gliomas), any MRI scan contraindications (presence of nonremovable metal including braces, severe claustrophobia), premature birth (gestational age < 34 weeks), birth weight < 2000 g. Based on these exclusion criteria, 15 children with NS or NSML and 20 TD individuals were excluded. Two individuals with previously documented FSIQ scores below 70 were excluded during screening. As shown in Table 2, cognitive scores varied within groups, particularly among those with *PTPN11*-associated NS, highlighting the range of abilities represented in the sample. To address potential bias from the higher average IQ in our TD sample, we removed TD individuals with IQ scores more than three standard deviations above the mean to ensure a more representative comparison group for our analyses. Notably, no participants in other groups scored above this threshold.

Before participation, written consent was obtained from the legal guardians of all participants, and participants over the age of 7 provided written assent. The neurodevelopmental, cognitive, and behavioral profiles of a subset of individuals in the current group have been previously reported [9, 10, 14, 40–42]. Expanding beyond *PTPN11* and *SOS1*-associated NS, we assessed behavioral outcomes across a broader range of NS- and NSML-causing genes. Additionally, we investigated whether biochemical markers of NS-associated pathogenic *PTPN11* variants are associated with distinct behavioral outcomes.

### Behavioral assessments

We administered age-appropriate versions of the Wechsler Intelligence Scales to measure global cognitive ability [43, 44]. Both the Wechsler Intelligence Scale for Children (WISC) and Wechsler Abbreviated Scale of Intelligence (WASI) provided Full-Scale IQ (FSIQ), Verbal IQ (VIQ), and Performance IQ (PIQ) scores. However, for a supplementary analysis, we used the FSIQ-2, which is derived only from the WASI, as it provided the most complete data across participants. Further details are provided in the Supplementary Materials.

We assessed social skills using the Social Responsiveness Scale Second Edition (SRS-2), a comprehensive 65-item parent-report questionnaire designed to evaluate the severity of behaviors related to social impairment and ASD-related behaviors [45]. We use ‘ASD-related behavior’ to describe the trait characteristics of ASD that may be present in an individual without a formal diagnosis. We utilized the composite SRS-2 total score in addition to the five SRS-2 subscales: social motivation, social awareness, social cognition, social communication, restricted interests, and repetitive behavior. To aid interpretation, we used the established categorical threshold labels provided by the SRS-2, which classify T-scores as follows: scores 59 and below are considered within the average range, 60–65 indicate mild range, 66–75 represent moderate range, and scores of 76 and above are considered in the severe range for ASD-related behaviors [46, 47]. Furthermore, to evaluate psychiatric disorders in children, we utilized the Kiddie Schedule for Affective Disorders and Schizophrenia for DSM-5 (KSADS), a semi-structured diagnostic interview widely used in clinical and research settings to assess current and past episodes of psychopathology in children and adolescents based on DSM criteria [48]. Although we did not obtain formal clinical ASD diagnoses through standardized diagnostic assessments, the KSADS provided broader psychiatric characterization. Three participants in our cohort had an ASD diagnosis: one with a prior community-based diagnosis and two who met criteria through the KSADS interview. An additional 13 participants endorsed more than four ASD-related symptoms on the KSADS.

Additionally, we assessed behavioral and emotional problems using the parent-report Child Behavior Checklist (CBCL) [49]. The seven problem scales within the CBCL included anxious/depressed, withdrawn/depressed, somatic complaints, thought problems, attention problems, rule-breaking behavior, and aggressive behavior. To aid interpretation, we used the established categorical thresholds provided by the CBCL, which classify T-scores as follows: scores between 50 and 64 fall within the average range, 65–69 suggest potential areas of concern or risk, and scores of 70 or higher indicate clinical levels of behavioral and emotional challenges [50–52].

### In vitro phosphatase assay

The human full-length polyHis-tagged *PTPN11* cDNA was cloned in a pET-26b vector (Novagen, Madison, WI). Single-base changes resulting in each of the identified missense changes were introduced by site-directed mutagenesis (QuikChange site-directed mutagenesis kit, Stratagene, San Diego, CA). Bi-directional Sanger sequencing confirmed the coding sequence of all constructs.

Recombinant proteins were expressed as previously described [53], using E. coli (DE3) Rosetta2 competent cells (Novagen, Madison, WI). Briefly, following induction with isopropyl β-D-1-thiogalactopyranoside (IPTG) (Roche, Rotkreuz, Switzerland) (2 h at 30 °C), harvesting, and cell lysis, polyHis-tagged SHP2 proteins were purified by chromatography, using nickel-nitrilotriacetic acid magnetic agarose beads (Qiagen, Hilden, Germany), and stored at -20 °C in the presence of 5 mM DTT (Sigma-Aldrich, St. Louis, MO).

SHP2 catalytic activity was evaluated using 200 ng of recombinant proteins and 20 mM p-nitrophenyl phosphate (pNPP) (Sigma-Aldrich, St. Louis, MO) as substrate, either basally or in the presence of the protein tyrosine phosphatase non-receptor type substrate 1 (PTPNS1) bisphosphotyrosyl-containing motif (hereafter, BTAM peptide) (GGGGDIT(pY)ADLNLPKGKKPAPQAAEPNNHTE(pY)ASIQTS) (Caslo, Kongens Lyngby, Denmark), as previously described [36]. Proteins were incubated for 30 min at 30 °C. Phosphate release was determined by measuring absorbance at 405 nm. The amount, purity, and integrity of recombinant proteins were assessed by protein assay kit (BioRad, Hercules, CA) and Coomassie Blue staining. Values are expressed as means of at least three independent experiments and are normalized to the basal activity of wild-type SHP2. The location of the SHP2 residues found mutated in the NS and NSML groups was analyzed by using the structure AF-Q06124-F1-v4, available in the AlphaFold Protein Structure Database (https://alphafold.ebi.ac.uk/entry/Q06124) [54].

To create classifications within the NS and NSML-associated *PTPN11* variants, we grouped individuals based on the location of the amino acid substitutions within SHP2. Class A (N = 78) includes residues involved in the N-SH2/PTP domain interface. Class B (N = 7) comprises residues implicated in protein–protein interactions, specifically, Thr42 in the N-SH2 domain and Glu139 in the C-SH2 domain. Class C (N = 3) contains residues located within the linker region connecting the two SH2 domains, exemplified by Asp106. Class D (N = 7) consists of residues mutated in NSML, including Tyr279, Gly464, and Thr468.

## Statistical analysis

All statistical analyses were performed in R v4.4.1 (R Core Team, 2024).

### Descriptive analysis

We conducted t-tests and Chi-square tests to examine differences between groups (NS/NSML vs. TD individuals) across demographic measures, including age, sex, full-scale intellectual quotient (FSIQ), verbal IQ (VIQ), and performance IQ (PIQ). For individual subgroups*,* the mean and range of scores for each behavioral outcome were obtained. For genetic variants that had one participant, we reported the individual's score. Moreover, we report the number and percentage of individuals within each genetic variant group, as well as TD individuals who fall one or two standard deviations (SD) below or above the mean for each IQ measure.

In addition, we report the prevalence of individuals within each genetic variant group who fall into clinically meaningful ranges, based on the normative thresholds outlined by the SRS-2 and CBCL. Specifically, we report the proportion of individuals categorized as “mild,” “moderate,” or “severe” for SRS-2 ASD-related traits, and “at-risk” or “clinical” for CBCL behavioral and emotional problems.

### Behavioral profiles

To examine the behavioral outcomes associated with each genetic variant group, we conducted between-group comparisons of the six SRS-2 and seven CBCL measures. As most of the data did not meet the assumption of normality, we employed non-parametric methods. This analysis was conducted only on TD individuals and the variant subgroups with at least five participants (*i.e.*, *PTPN11-*associated NS and NSML, *SOS1*, and *RAF1*).

We first compared each variant subgroup (*PTPN11-*associated NS and NSML, *SOS1*, and *RAF1*) to the TD individuals. For each behavioral measure (SRS-2 and CBCL), we performed a Kruskal–Wallis test to assess whether there were overall significant differences. The Kruskal–Wallis test was chosen as evidence suggests it better protects against inflated error rates compared to parametric alternatives, particularly in the cases of small sample sizes [55]. Furthermore, the Kruskal–Wallis is often used in genotype–phenotype analyses [56, 57]. When the Kruskal–Wallis test indicated a significant effect (*p* < 0.05), we performed post-hoc pairwise comparisons between each variant subgroup and TD using Dunn’s test. To control for multiple comparisons, the resulting p-values from Dunn’s tests were adjusted using the Benjamini-Hochberg (BH) false discovery rate (FDR) correction, applied separately within each behavioral assessment (SRS-2 and CBCL).

Following this, we excluded the TD group and conducted a secondary exploratory analysis to compare the genetic variant groups to each other directly. This analysis focused on the four largest groups: *PTPN11*-associated NS and NSML, *SOS1*, and *RAF1*. As before, Kruskal–Wallis tests were used to assess group differences, followed by post-hoc Dunn’s tests with BH FDR correction when significant results were found*.*

Across both analyses, we report Cliff’s Delta (δ) as a non-parametric measure of effect size to contextualize the magnitude of clinical significance of group differences. Cliff’s Delta quantifies the degree of overlap between two groups, providing a robust estimate of the magnitude of difference regardless of sample size or distribution. We adopt a modest approach to interpret Cliff’s Delta values based on conventional thresholds: values of 0.147, 0.33, and 0.474 represent small, medium, and large effects, respectively [58].

To assess potential sex differences, we conducted Mann–Whitney U tests within each diagnostic group with at least five individuals per sex (e.g., TD individuals, individuals with *PTPN11*-associated NS, and individuals with *SOS1*). Analyses were conducted across all SRS-2 subscales (measuring social responsiveness and ASD-related traits) and CBCL subscales (measuring emotional and behavioral problems). Additional information regarding the statistical analysis can be found in the Supplementary Methods. Furthermore, to assess whether differences in IQ influence behavioral scores, we conducted an additional analysis using behavioral scores from the SRS-2 and CBCL residualized for IQ (see Supplementary Methods).

### Genotype–phenotype analyses: biochemical predictors of behavior in NS-associated PTPN11 variants

We assessed potential correlations between SHP2’s basal activity, stimulated catalytic activity, and fold activation with behavior measured by SRS-2 and CBCL. We conducted logistic regression analyses within the *PTPN11*-associated NS variant subgroup to calculate odds ratios (ORs), quantifying the likelihood of belonging to the severe group for each unit increase in a biochemical parameter. The behavioral outcomes included all SRS-2 and CBCL measures except SRS-2 social motivation due to low variability. For these analyses, we set a threshold defined as a T score > 75 (2.5 SD above the mean) across both age and sex-corrected SRS-2 and CBCL measures. Each model examined a single behavioral outcome against one biochemical predictor. This analysis provides insights into the degree of impairment related to social behavior, rather than focusing on diagnostic labels.

## Results

### Demographic characteristics

The combined NS and NSML group compared to the TD group did not differ in age (*p* = 0.974) or sex (*p* = 0.092) (Table 1)*.* Compared to TD individuals, affected individuals had significantly lower scores for the FSIQ-2 (*p* = 6.45 × 10^–9^), VIQ (*p* = 6.31 × 10^–15^), and PIQ (*p* = 1.30 × 10^–13^) scales. A detailed breakdown of IQ score distributions across diagnostic thresholds and genetic variant subgroups is available in the Supplementary Results and Table S1. Demographic and descriptive information, including average scores in the SRS-2 and CBCL subscales for each variant subgroup, is presented in Table 2. Of the groups with five or more participants, *PTPN11-*associated NSML demonstrated the highest average scores on all SRS-2 and CBCL measures except for rule-breaking behavior. Conversely, the *RAF1*-associated NS subgroup scored the lowest on all SRS-2 and CBCL measures except for the withdrawn/depressed subscale.

### Variant-specific heterogeneity in ASD-related traits and behavioral and emotional problems compared to a normative sample

Individuals with NS and NSML show substantial heterogeneity in ASD-related traits and behavioral and emotional problems across variant subgroups (Table 3). While average scores on the SRS-2 and CBCL are often within the mild or moderate range, suggesting deficiencies in social behavior that may or may not interfere with daily functioning, a notable subset, particularly those with *PTPN11*-associated NS, NSML, and *SOS1* variants, show moderate to severe impairment in domains such as restricted and repetitive behaviors and social cognition. In contrast, most TD individuals scored within the average range across all domains, as did those with *RAF1* variants. Behavioral and emotional problems also varied by variant subgroup, with *PTPN11*-associated NS and NSML subgroups showing the highest rates of elevated symptoms, especially in anxiety, attention, and somatic complaints. Whereas those with the *SOS1* and *RAF1* variant subgroups and TD individuals had fewer clinically elevated scores for behavioral and emotional problems.

**Table 3:** Prevalence of Behavioral and Cognitive Phenotype Categories by Diagnosis in Individuals with NS and NSML-Causing Variants and TD Individuals

	**TD (N = 71)**	***PTPN11*-NS (N = 88)**	***SOS1-*NS** **(N = 18)**	***PTPN11-*NSML** **(N = 7)**	***RAF1-*NS** **(N = 6)**
**SRS-2 Measures, n (%)**					
*Social responsiveness total*					
Average	62 (88.6%)	39 (44.3%)	10 (55.6%)	1 (14.3%)	5 (83.3%)
Mild	4 (5.7%)	18 (20.5%)	2 (11.1%)	2 (28.6%)	0 (0%)
Moderate	4 (5.7%)	21 (23.9%)	4 (22.2%)	4 (57.1%)	0 (0%)
Severe	0 (0%)	10 (11.4%)	2 (11.1%)	0 (0%)	1 (16.7%)
*Restricted and repetitive behaviors*					
Average	65 (92.9%)	42 (47.7%)	9 (50%)	2 (28.6%)	5 (83.3%)
Mild	2 (2.9%)	11 (12.5%)	1 (5.6%)	2 (28.6%)	0 (0%)
Moderate	2 (2.9%)	20 (22.7%)	5 (27.8%)	2 (28.6%)	0 (0%)
Severe	1 (1.4%)	15 (17%)	3 (16.7%)	1 (14.3%)	1 (16.7%)
*Social cognition*					
Average	64 (91.4%)	48 (54.5%)	10 (55.6%)	3 (42.9%)	5 (83.3%)
Mild	5 (7.1%)	15 (17%)	4 (22.2%)	0 (0%)	0 (0%)
Moderate	1 (1.4%)	17 (19.3%)	3 (16.7%)	4 (57.1%)	0 (0%)
Severe	0 (0%)	8 (9.1%)	1 (5.6%)	0 (0%)	1 (16.7%)
*Social communication*					
Average	62 (88.6%)	43 (48.9%)	11 (61.1%)	2 (28.6%)	5 (83.3%)
Mild	4 (5.7%)	15 (17%)	0 (0%)	1 (14.3%)	0 (0%)
Moderate	4 (5.7%)	19 (21.6%)	6 (33.3%)	4 (57.1%)	0 (0%)
Severe	0 (0%)	11 (12.5%)	1 (5.6%)	0 (0%)	1 (16.7%)
*Social motivation*					
Average	61 (87.1%)	54 (61.4%)	13 (72.2%)	3 (42.9%)	5 (83.3%)
Mild	5 (7.1%)	17 (19.3%)	2 (11.1%)	1 (14.3%)	0 (0%)
Moderate	3 (4.3%)	13 (14.8%)	2 (11.1%)	2 (28.6%)	1 (16.7%)
Severe	1 (1.4%)	4 (4.5%)	1 (5.6%)	1 (14.3%)	0 (0%)
**CBCL Measures, n (%)**					
*Anxious/depressed*					
Average	60 (87%)	11 (12.5%)	16 (88.9%)	4 (57.1%)	5 (83.3%)
At-Risk	8 (11.6%)	68 (77.3%)	0 (0%)	0 (0%)	0 (0%)
Clinical	1 (1.4%)	9 (10.2%)	2 (11.1%)	3 (42.9%)	1 (16.7%)
*Withdrawn/depressed*					
Average	64 (92.8%)	73 (83%)	17 (94.4%)	6 (85.7%)	6 (100%)
At-Risk	4 (5.8%)	11 (12.5%)	0 (0%)	0 (0%)	0 (0%)
Clinical	1 (1.4%)	4 (4.5%)	1 (5.6%)	1 (14.3%)	0 (0%)
*Somatic complaints*					
Average	66 (95.7%)	67 (76.1%)	12 (66.7%)	2 (28.6%)	6 (100%)
At-Risk	2 (2.9%)	14 (15.9%)	4 (22.2%)	1 (14.3%)	0 (0%)
Clinical	1 (1.4%)	7 (8%)	2 (11.1%)	4 (57.1%)	0 (0%)
*Thought problems*					
Average	61 (88.4%)	54 (61.4%)	10 (55.6%)	2 (28.6%)	5 (83.3%)
At-Risk	6 (8.7%)	17 (19.3%)	5 (27.8%)	2 (28.6%)	0 (0%)
Clinical	2 (2.9%)	17 (19.3%)	3 (16.7%)	3 (42.9%)	1 (16.7%)
*Attention problems*					
Average	61 (88.4%)	50 (56.8%)	14 (77.8%)	1 (14.3%)	4 (66.7%)
At-Risk	3 (4.3%)	24 (27.3%)	2 (11.1%)	4 (57.1%)	2 (33.3%)
Clinical	5 (7.2%)	14 (15.9%)	2 (11.1%)	2 (28.6%)	0 (0%)
*Rule-breaking behavior*					
Average	65 (94.2%)	75 (85.2%)	14 (77.8%)	7 (100%)	6 (100%)
At-Risk	4 (5.8%)	8 (9.1%)	4 (22.2%)	0 (0%)	0 (0%)
Clinical	0 (0%)	5 (5.7%)	0 (0%)	0 (0%)	0 (0%)
*Aggressive behavior*					
Average	43 (81.1%)	74 (85.1%)	14 (82.4%)	4 (57.1%)	5 (83.3%)
At-Risk	6 (11.3%)	8 (9.2%)	1 (5.9%)	1 (14.3%)	1 (16.7%)
Clinical	4 (7.5%)	5 (5.7%)	2 (11.8%)	2 (28.6%)	0 (0%)

NS, Noonan syndrome; NSML, Noonan syndrome with multiple lentigines; TD, typically developing individuals; SRS-2, Social Responsiveness Scale Second Edition; CBCL, Child Behavior Checklist

Higher scores for the SRS-2 and CBCL indicate clinically elevated symptoms and greater impairment. Data show the number and percentage of individuals within each variant subgroup categorized by severity level on the SRS-2 and CBCL measures. Percentages reflect the proportion within each variant group falling in the respective category

### Variant-specific differences in ASD-related traits, behavioral, and emotional problems compared to TD individuals

**Fig. 1:**
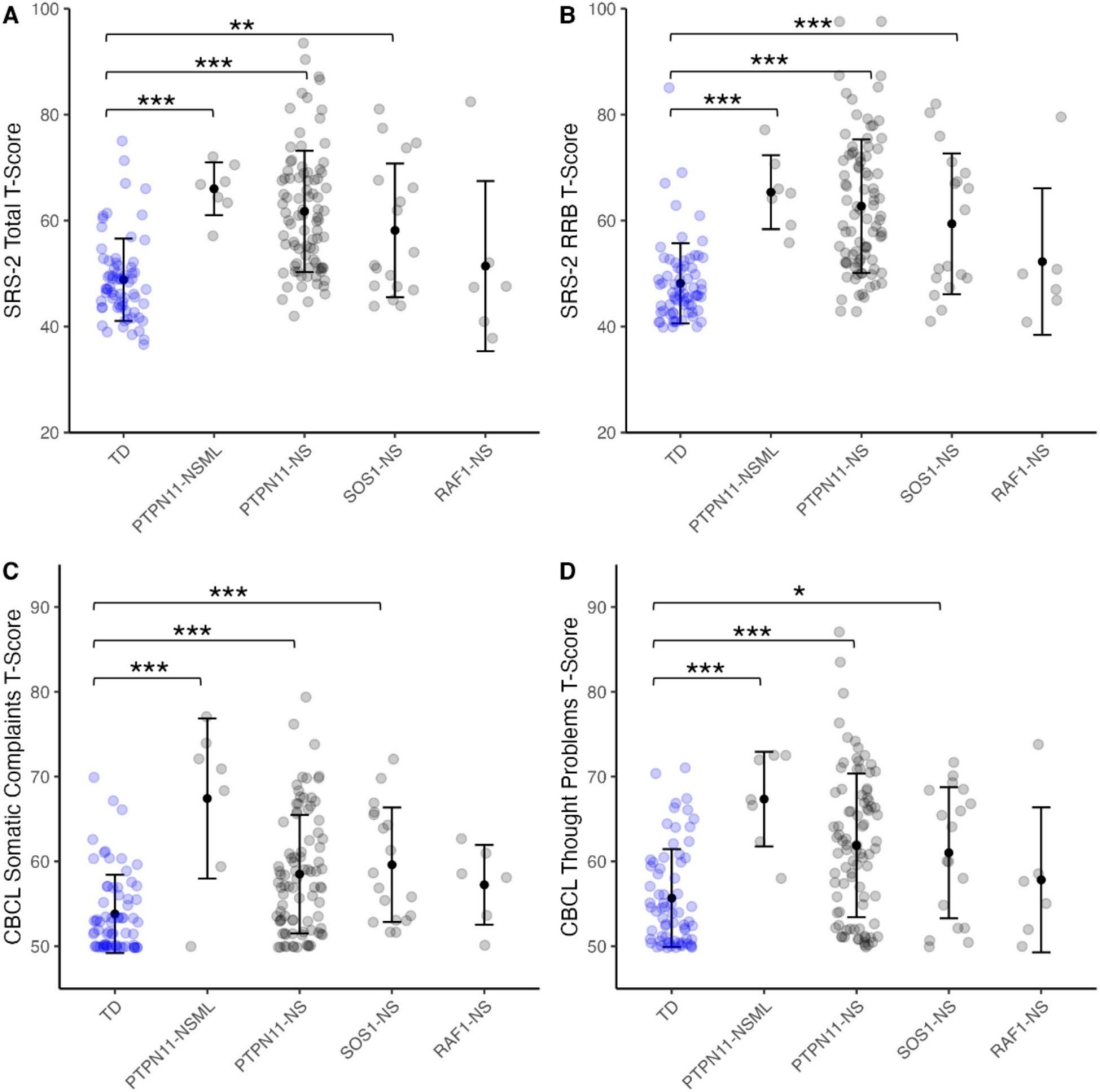
Social impairment and behavioral challenges in the study cohorts. Relative to TD individuals (blue), specific variant subgroups exhibit greater social impairment and behavioral challenges. Compared to TD individuals, all subgroups but the *RAF1*-associated NS subgroup showed elevated (A) SRS-2 total T-scores, (B) SRS-2 RRB T-scores, (C) CBCL somatic complaints T-scores, and (D) CBCL thought problems T-scores. The *RAF1*-associated NS subgroup did not differ significantly from TD individuals on any SRS-2 or CBCL measures. ***indicates FDR-corrected *p*-value < .001, **p-value < .010, *p-value < .050. Points and error bars indicate means and standard deviations, respectively. *NS, Noonan syndrome; NSML, Noonan syndrome with multiple lentigines; TD, typical developing; SRS-2, Social Responsiveness Scale Second Edition; CBCL, Child Behavior Checklist*

We observed distinct patterns of behavioral and emotional problems, with specific variant subgroups exhibiting unique profiles. Specifically, pronounced differences were found when considering social responsiveness, emotional problems, and social competence from the SRS-2 (Fig. 1A–1B; Table 4). Group comparisons using the Kruskal–Wallis test revealed significant differences across genetic variants and TD individuals on all SRS-2 subscales (*p*_*uncorrected*_ < 0.001) and all CBCL subscales (*p*_*uncorrected*_ < 0.05), except for withdrawn/depressed behavior and rule-breaking behavior, which were not significant (Table 4). These results suggest broad behavioral differences across groups, particularly in social responsiveness and behavioral and emotional problems. Post hoc comparisons using Dunn’s tests revealed significant differences in social responsiveness across all the NS and NSML subgroups when compared to TD individuals, suggesting increased ASD-related behaviors in these groups. Additionally, large effect sizes were found, emphasizing the clinical significance of these differences. Specifically, compared to TD individuals, those with *PTPN11-*associated NS (Cliff’s δ > 0.62; *p*_*FDR*_ < 0.001), *PTPN11-*associated NSML (Cliff’s δ > 0.84; *p*_*FDR*_ < 0.001), and *SOS1-*associated NS (Cliff’s δ > 0.38; *p*_*FDR*_ = 0.015–0.001) showed significantly elevated scores in social responsiveness total scores, restricted and repetitive behaviors, social cognition, and social communication (Fig. 1A–B; Table 4). These findings indicate clinically significant impairments and elevated levels of ASD-related behaviors, underscoring the pronounced social and behavioral challenges associated with these genetic variants and distinguishing them from those seen in TD individuals.

**Table 4:** Dunn Test Results Between Genetic Variants Compared to Typically Developing Individuals

	Kruskal–Wallis	*PTPN11-*NS vs. TD		*PTPN11-*NSML vs. TD		*SOS1*-NS vs. TD		*RAF1*-NS vs. TD	
	*P-value*	*P*_*FDR*_-value	Effect Size	*P*_*FDR*_-value	Effect Size	*P*_*FDR*_-value	Effect Size	*P*_*FDR*_-value	Effect Size
*SRS-2 measures*									
Social responsiveness total	** < 0.001**	** < 0.001**	0.685	** < 0.001**	0.898	**0.004**	0.453	0.388	−0.024
Restricted and repetitive behaviors	** < 0.001**	** < 0.001**	0.730	** < 0.001**	0.908	** < 0.001**	0.521	0.248	0.143
Social cognition	** < 0.001**	** < 0.001**	0.658	** < 0.001**	0.904	**0.004**	0.435	0.392	−0.014
Social communication	** < 0.001**	** < 0.001**	0.624	** < 0.001**	0.847	**0.015**	0.383	0.333	0.021
Social motivation	** < 0.001**	** < 0.001**	0.388	**0.004**	0.676	0.129	0.196	0.480	0.024
*CBCL measures*									
Anxious/depressed	**0.009**	**0.039**	0.224	**0.007**	0.737	0.320	0.120	0.480	−0.010
Somatic complaints	** < 0.001**	** < 0.001**	0.420	** < 0.001**	0.723	** < 0.001**	0.548	0.101	0.457
Thought problems	** < 0.001**	** < 0.001**	0.447	** < 0.001**	0.839	**0.016**	0.393	0.306	0.138
Attention problems	** < 0.001**	** < 0.001**	0.503	** < 0.001**	0.832	0.050	0.305	0.124	0.312
Aggressive behavior	**0.010**	**0.007**	0.294	**0.041**	0.484	0.211	0.217	0.361	0.143

NS, Noonan syndrome; NSML, Noonan syndrome with multiple lentigines; TD, typically developing individuals; SRS-2, Social Responsiveness Scale Second Edition; CBCL, Child Behavior Checklist

Bolded values represent significant *p*-values (*p* < .05)

Cliff’s delta effect sizes are reported, where a small effect size is δ = 0.147 to 0.330, a small medium effect is δ = 0.330 to 0.474, and a large effect is δ > 0.474

Bolded values represent significant *p*-values after FDR correction (*p* < .05)

Similar patterns were observed across CBCL measures, assessing behavioral and emotional problems, when compared to TD individuals. Kruskal–Wallis tests indicated significant group differences across all CBCL measures (*p* < 0.05), except for withdrawn/depressed behaviors and rule-breaking behavior (Table 4). Additionally, medium to large effect sizes were found, emphasizing the clinical significance of these behavioral differences; post-hoc Dunn tests showed that individuals with *PTPN11-*associated NS and NSML, and *SOS1-*associated NS variants exhibited significantly higher scores on both somatic complaints and thought problems compared to TD individuals (Table 4). Specifically, elevated levels were observed in somatic complaints and thought problems among individuals with *PTPN11*-associated NS (Somatic complaints: [Cliff’s δ = 0.42, *p* < 0.001]; Thought Problems: [Cliff’s δ = 0.45, *p*_*FDR*_ < 0.001]), *PTPN11-*associated NSML (Somatic complaints: [Cliff’s δ = 0.72, *p* = 0.007]; Thought Problems: [Cliff’s δ = 0.84, *p*_*FDR*_ < 0.001]), and *SOS1*-associated NS (Somatic complaints: [Cliff’s δ = 0.55; *p*_*FDR*_ < 0.001]; Thought Problems: [Cliff’s δ = 0.39, *p*_*FDR*_ = 0.016]) (Fig. 1C-1D; Table 4). These findings suggest a consistent pattern of increased internalizing symptoms across these subgroups. Additionally, significantly elevated scores in anxious/depressed behaviors and attention problems were specific to individuals with *PTPN11-*associated NS (Anxious/depressed: [Cliff’s δ = 0.22, *p*_*FDR*_ = 0.039]; Attention Problems: [Cliff’s δ = 0.50, *p*_*FDR*_ < 0.001]) and *PTPN11-*associated NSML (Anxious/depressed: [Cliff’s δ = 0.74, *p*_*FDR*_ = 0.007]; Attention Problems [Cliff’s δ = 0.83, *p*_*FDR*_ < 0.001]), with many participants falling within the at-risk or clinical range (Table 4). These results highlight a distinct pattern of heightened emotional and behavioral problems in individuals with *PTPN11*-associated NS and NSML. Uniquely, *PTPN11-*associated NS and NSML also showed significantly elevated scores in aggressive behavior compared to TD individuals (*PTPN11*-associated NS: Cliff’s δ = 0.29, *p*_*FDR*_ = 0.007; *PTPN11*-associated NSML: Cliff’s δ = 0.48, *p*_FDR_ = 0.041), highlighting additional challenges with behavioral regulation and impulsivity [59]. In contrast, withdrawn/depressed and rule-breaking behavior did not show significant group differences in the Kruskal–Wallis analysis, suggesting this domain may be less affected across these variant subgroups compared to TD individuals.

Furthermore, Mann–Whitney U tests of TD, *PTPN11*-associated NS, and *SOS1* groups revealed no significant sex differences on SRS-2 or CBCL measures (Table S2). Moreover, we observed that behavioral differences persisted after accounting for IQ. Kruskal–Wallis tests revealed significant group differences across all SRS-2 subscales, except for social motivation, as well as somatic complaints, thought problems, and attention problems measured by the CBCL (Table S3). Distinct profiles of ASD-related behaviors persisted in individuals with *PTPN11*-associated NS, *PTPN11*-associated NSML, and *SOS1* variants, while the *RAF1* variant subgroup did not differ from TD individuals. In contrast, CBCL differences were more sensitive to IQ adjustment, with fewer group-level effects remaining significant. However, significant emotional and behavioral problems continued to be observed in individuals with *PTPN11*-associated NSML and *SOS1* variants. Further results are provided in the Supplementary Materials (Table S3).

### Variant-Specific Differences in ASD-Related Traits, Behavioral, and Emotional Problems Across Variant Subgroups

Group comparisons using the Kruskal–Wallis test across genetic variant subgroups revealed limited significant differences in behavioral scores. For the SRS-2, significant group differences were observed in social responsiveness total score (*p*_*uncorrected*_ = 0.035, Table 5) and social cognition scores (*p*_*uncorrected*_ = 0.049, Table 5), while the remaining subscales did not reach significance (Table S4). Within the CBCL subscales, only attention problems showed a significant difference across groups (*p*_*uncorrected* =_ 0.032, Table 5), with anxious/depressed behaviors and somatic complaints approaching but not reaching significance (Table S4).

**Table 5:** Dunn Test Results after FDR-correction Between-Group Differences for the SRS-2 and CBCL Measures

	Kruskal–Wallis	*PTPN11-*NS vs. *PTPN11-*NSML	*PTPN11-*NS versus *RAF1*-NS	*PTPN11-*NSML versus *RAF1-*NS	*PTPN11-*NS versus *SOS1-*NS	*PTPN11-* NSML versus *SOS1-*NS	*SOS1-*NS versus *RAF1*-NS
	***P-*value**	**Effect Sizes**					
*SRS-2 measures*							
Social responsiveness total	**0.035**	-0.321	**0.561**	**0.667**	0.208	0.397	0.315
Social cognition	**0.049**	-0.351	0.534	**0.667**	0.157	0.389	0.361
*CBCL measures*							
Attention problems	**0.032**	**-0.544**	0.176	**0.690**	0.219	**0.683**	-0.046

NS, Noonan syndrome; NSML, Noonan syndrome with multiple lentigines; SRS-2, Social Responsiveness Scale Second Edition; CBCL, Child Behavior Checklist

Cliff’s delta effect sizes are reported, where a small effect size is δ = 0.147 to 0.330, a small medium effect is δ = 0.330 to 0.474, and a large effect is δ > 0.474

Bolded values represent significant *p*-values (*p* < .05)

**Fig. 2:**
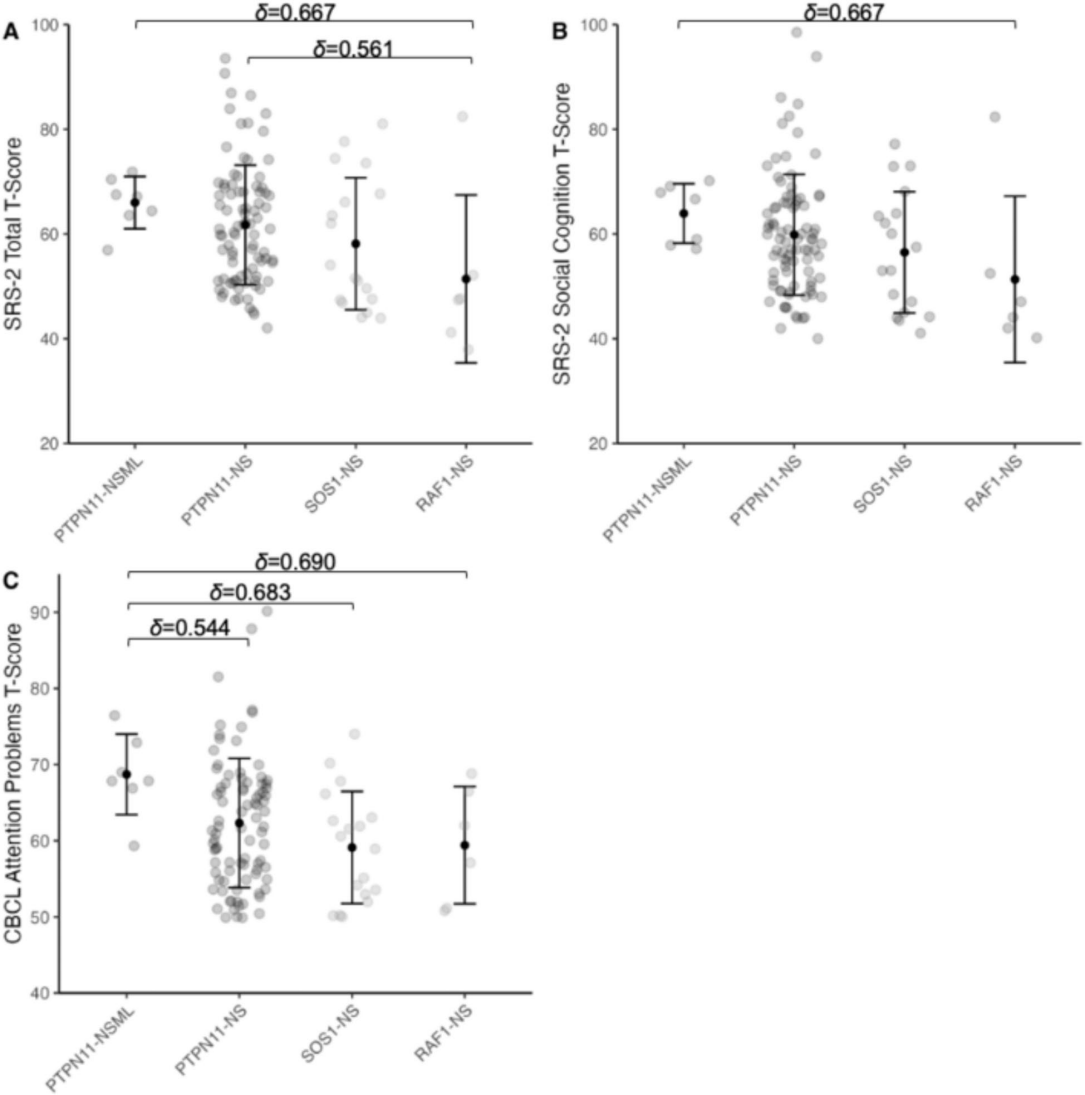
Comparisons between variant subgroups reveal differences in social and behavioral outcomes. **A** Elevated SRS-2 total t-scores in the *PTPN11*-associated NS and NSML subgroups relative to *RAF1*-associated NS. **B** Elevated SRS-2 social cognition t-scores in the *PTPN11*-associated NSML subgroup relative to *RAF1*-associated NS. **C** Elevated CBCL attention problems t-scores in the *PTPN11*-associated NSML subgroup relative to the *PTPN11-, SOS1-*, and *RAF1-*associated NS subgroups. Cliff’s delta effect sizes are reported in the abbreviated line for measures that reached significance after FDR correction (*p* < .05). Points and error bars indicate means and standard deviations, respectively. *NS, Noonan syndrome; NSML, Noonan syndrome with multiple lentigines; SRS-2, Social Responsiveness Scale Second Edition; CBCL, Child Behavior Checklist*

Importantly, post-hoc Dunn tests incorporating Cliff’s delta effect sizes, which represent the magnitude of clinical significance, revealed that individuals with *PTPN11*-associated NS (Cliff’s δ = 0.56, *p*_*FDR*_ = 0.040) and *PTPN11*-associated NSML (Cliff’s δ = 0.67, *p*_*FDR*_ = 0.030) had higher social responsiveness total scores compared to those with *RAF1* variants (Fig. 2A, Table 5). Large effect sizes were also observed for social cognition, with *PTPN11*-associated NSML showing elevated scores relative to the *RAF1* variant subgroup (Cliff’s δ = 0.67; *p*_*FDR*_ = 0.027) (Fig. 2B, Table 5). These findings highlight heightened social difficulties associated with this class of *PTPN11* variants and provide initial insight into genetic variant-specific behavioral profiles within NS and NSML.

CBCL results further illustrated areas of behavioral heterogeneity within specific genetic variant subgroups. Large effect sizes were observed, specifically, individuals with *PTPN11*-associated NSML had significantly higher attention problems (*p* < 0.03) compared to all other genetic variant subgroups (Fig. 2C, Table 5). These effect sizes suggest modest to large clinical differences, providing initial evidence of behavioral heterogeneity amongst the distinct genetic variant subgroups causing NS and NSML.

While differences were modest and largely exploratory, these subgroups consistently showed mild to severe scores in ASD-related behaviors as measured by the SRS-2. The observed clinical heterogeneity, reflected in the magnitude of the Cliff’s delta values, highlights the importance of continued, more detailed investigation to better understand and ultimately tailor interventions for the diverse behavioral manifestations associated with NS and NSML.

### Biochemical Predictors of Behavior in Individuals with NS-associated *PTPN11* Variants

**Fig. 3:**
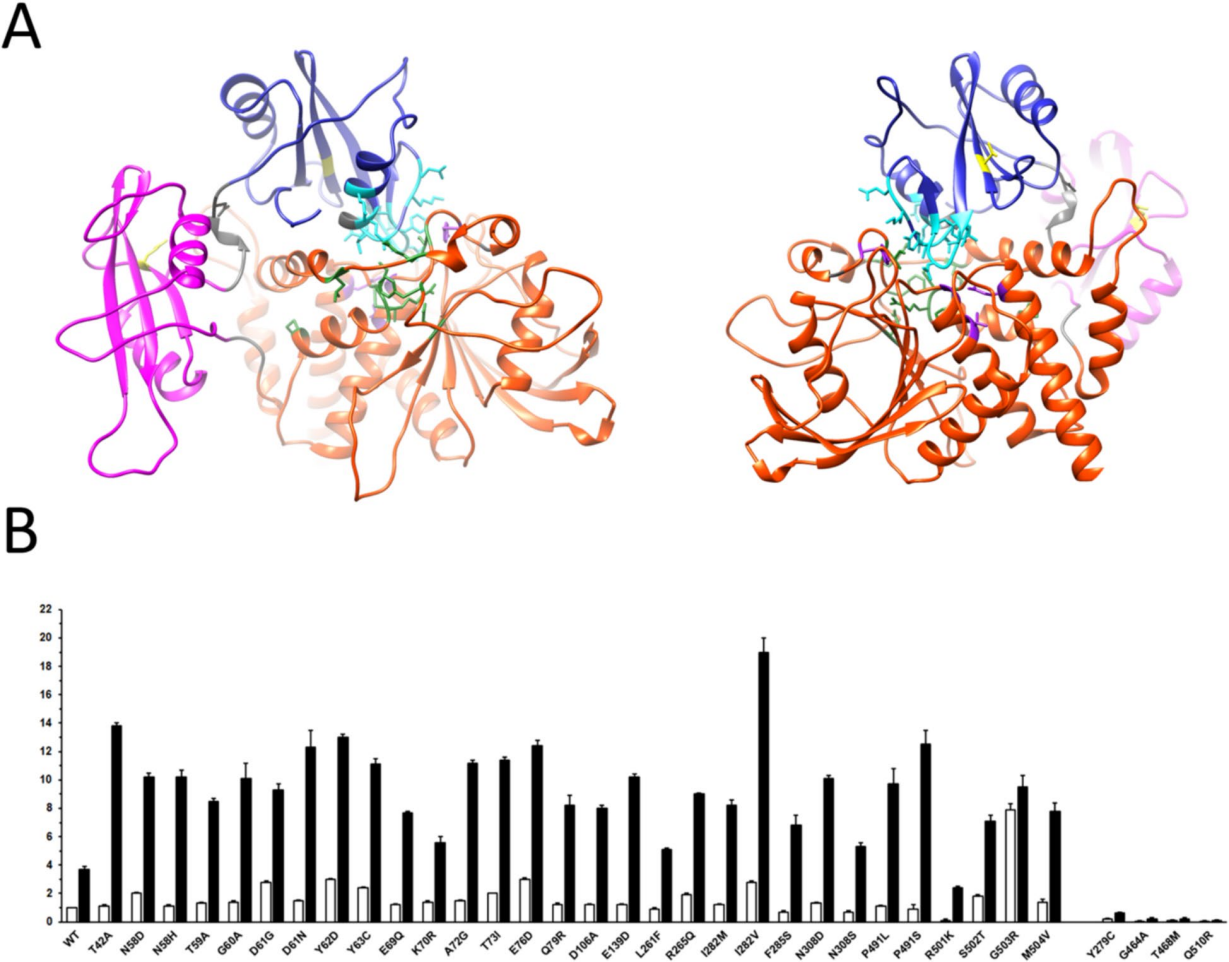
Location of affected residues in the catalytically inactive conformation of SHP2 and in vitro catalytic activity of tested mutants. **A** Residues mutated in individuals with *PTPN11*-associated NS and NSML are shown with their lateral chains. Residues involved in the N-SH2/PTP interaction are shown in cyan (N-SH2 domain) and green (PTP domain); residues implicated in binding to partners (Thr42, N-SH2 domain; Glu139, C-SH2 domain) are in yellow; Asp106, which is located in the linker connecting the two SH2 domain is shown in black; residues mutated in *PTPN11*-associated NSML (Tyr279, Gly464, Thr468 and Gln510) are in violet. The three major domains (N-SH2, blue; C-SH2, pink; PTP, orange) are shown using two orthogonal views of SHP2 (AF-Q06124-F1-v4).**B** In vitro assessment of the phosphatase activity of recombinant SHP2 proteins, basally and following BTAM stimulation. Catalytic activity is compared with that of wild-type SHP2. Phosphatase activity was measured as pmoles of phosphate released using pNPP as a substrate, basally (white bars) and following stimulation with 10 μM BTAM peptide (black bars). Statistical differences compared to basal, stimulated activities of the WT enzyme, and fold activation are reported in Table 6 and Table S6

To further understand the variability in ASD-related traits, as well as behavioral and emotional problems, we analyzed our largest group of individuals with *PTPN11*-associated NS to investigate how the biochemical behavior of identified mutants contributes to the observed clinical heterogeneity. Specifically, we assessed the basal and stimulated catalytic activity of SHP2 mutants, as well as their fold activation; detailed values are provided in the Supplementary Materials (Table S5; Fig. 3).

### Relationships Between Catalytic Activity and ASD-Related Traits and Behavioral and Emotional Problems in *PTPN11*-associated NS

**Fig. 4:**
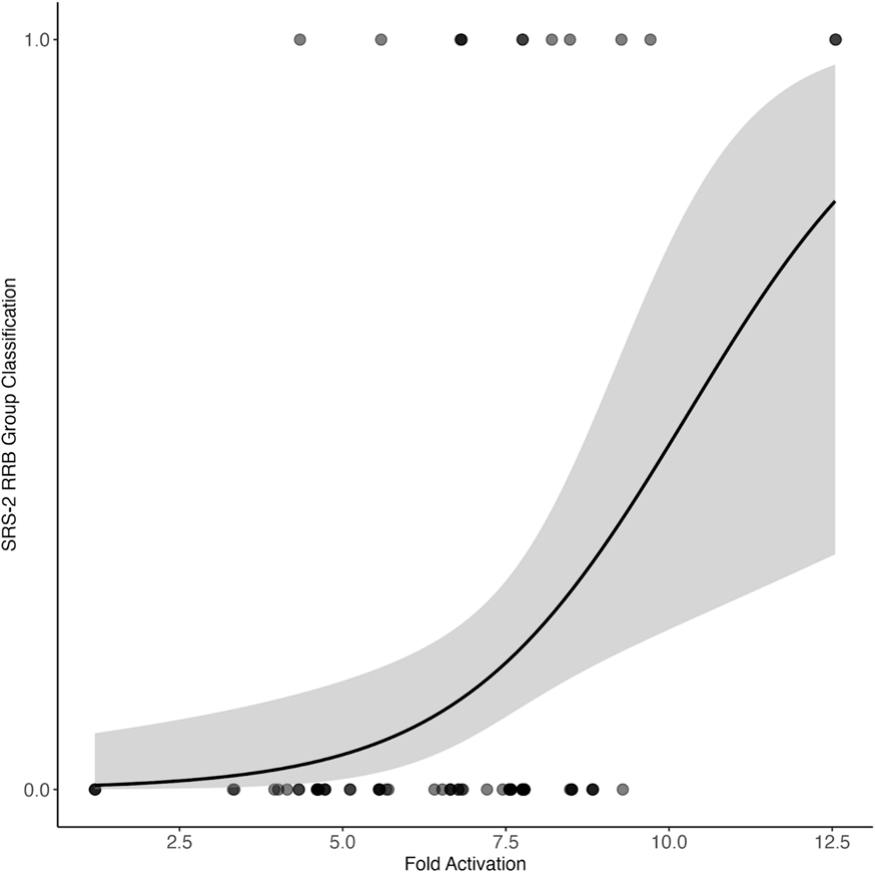
Logistic model depicting the relationship between fold activation in individuals with *PTPN11*-associated NS and restricted and repetitive behaviors from SRS-2. Gray circles represent individual data points (note that circles appear darker if there is overlap of data points), the solid black line shows the fitted logistic curve, and the gray shading indicates the 95% confidence interval. Fold activation is the ratio of stimulated phosphate release to basal activity (i.e., the relative increase in enzyme activity in stimulated compared to basal conditions). *NS, Noonan syndrome; SRS-2, Social Responsiveness Scale Second Edition*

Moreover, in our logistic regression models with SRS-2 measures as outcomes, we observed no significant differences when examining the basal (i.e., unstimulated) or stimulated catalytic activity of mutants (Table 6; Table S6). However, fold activation emerged as a significant predictor of elevated restricted and repetitive behaviors (*p*_*FDR*_ = 0.028, odds ratio = 1.64, 95% CI [1.19–2.49]), as documented by a logistic regression model using SRS-2 measures as outcomes (Fig. 4; Table 6). Specifically, each one-unit increase in fold activation was associated with a 64% increase in the odds of classification in the more severe group for restricted and repetitive behaviors. Although other SRS-2 measures did not reach significance, exploratory patterns for fold activation were observed for social responsiveness total score (*p*_FDR=_ 0.089; odds ratio = 1.40, 95% CI [1.00–2.05]) and social cognition (*p*_FDR=_ 0.089; odds ratio = 1.43, 95% CI [0.99–2.16]).

**Table 6:** The Logistic Regression Results for the SRS-2 and CBCL Measures for *PTPN11*-associated NS Variants

	Stimulated phosphatase release activity			Fold activation		
	Odds Ratio	*P*_uncorrected_	*P*_FDR-corrected_	Odds Ratio	*P*_uncorrected_	*P*_FDR-corrected_
*SRS-2 Measures*						
Social Responsiveness Total	1.21[0.97–1.51]	0.081	0.193	1.40[1.00–2.05]	0.061	0.089
Restricted and Repetitive Behaviors	1.12[0.92–1.37]	0.228	0.305	1.64[1.19–2.49]	**0.007**	**0.028**
Social Cognition	1.13[0.87–1.42]	0.315	0.315	1.43[0.99–2.16]	0.067	0.089
Social Communication	1.19[0.96–1.48]	0.096	0.193	1.18[0.86–1.63]	0.316	0.316
*CBCL measures*						
Anxious/depressed	1.23[0.85–1.67]	0.212	0.566	0.44[0.20–0.79]	**0.014**	0.097
Withdrawn/depressed	0.92[0.50–1.45]	0.772	0.772	1.31[0.67–2.52]	0.421	0.589
Somatic complaints	0.88[0.47–1.42]	0.672	0.772	1.33[0.68–2.56]	0.395	0.589
Thought problems	1.18[0.84–1.57]	0.276	0.566	1.38[0.84–2.28]	0.200	0.513
Attention problems	1.17[0.86–1.52]	0.263	0.566	0.97[0.63–1.50]	0.881	0.886
Rule-breaking Behavior	0.79[0.28–1.55]	0.582	0.772	1.07[0.42–2.65]	0.886	0.886
Aggressive Behavior	1.18[0.80–1.62]	0.323	0.566	0.71[0.41–1.23]	0.220	0.513

SRS-2: Social Responsiveness Scale Second Edition; CBCL, Child Behavior Checklist

Bolded values represent significant* p*-values (*p* < .05)

In logistic regression models with CBCL measures as outcomes, none of the biochemical predictors remained significant after FDR correction. Nonetheless, initial analyses indicated that increased fold activation was associated with a 56% reduction in the odds of predicting the clinical group of CBCL anxious/depressed behavior (*p*_uncorrected=_ 0.014, odds ratio = 0.44, 95% CI [0.20–0.79]) (Table 6). Lastly, basal activity indicated that increased activity was associated with an 84% increase in the odds of being classified in the severe group for anxious/depressed behavior (*p*_uncorrected_ = 0.007, odds ratio = 1.84, 95% CI [1.16–3.01]) (Table S6). However, these results did not survive FDR correction.

## Discussion

In the present study, we examined the behavioral variability characterizing NS and NSML and the biochemical patterns contributing to genotype–phenotype relationships in individuals with NS due to *PTPN11* variants. While previous research has predominantly focused on NS, with *PTPN11* and *SOS1* variants, we provide data considering the largest *PTPN11* group to date and include individuals with *PTPN11*-associated NSML and *RAF1*-associated NS variants, offering deeper insights into the behavioral profiles of these populations. Individuals with *PTPN11-*associated NS and NSML and *SOS1*-associated NS demonstrated clinically elevated scores on SRS-2 and CBCL measures, reflecting increased ASD-related behaviors, poorer social functioning, and increased emotional problems compared to TD individuals. A consistent pattern of impairment level emerged among the analyzed subgroups: *PTPN11*-associated NSML > *PTPN11*-associated NS > *SOS1*-associated NS > *RAF1*-associated NS. *PTPN11-*associated NS and NSML groups were particularly associated with heightened ASD-related behaviors, emotional problems, and attention problems. Genetic variant comparisons suggest differences in ASD-related behaviors, with individuals with *PTPN11*-associated NS and NSML exhibiting greater challenges compared to those with *RAF1*. Furthermore, our genotype–phenotype analysis provided the first evidence of the contribution of SHP2’s catalytic activity to the phenotypic heterogeneity associated with *PTPN11*-associated NS, particularly in restricted and repetitive behaviors. Logistic regression demonstrated that for each one-unit increase in fold activation was linked to a 64% higher likelihood of belonging to the severe range of restricted and repetitive behaviors group. These findings underscore the biological underpinnings of behavioral variability in NS and NSML and highlight the potential for targeted interventions based on genetic variant-specific risk profiles.

Compared to TD individuals, NS and NSML groups showed significant differences in ASD-related symptoms across multiple domains of social communication and responsiveness, as measured by the SRS-2. On average, individuals with *PTPN11*-associated NSML, *PTPN11-*associated NS*,* and *SOS1*-associated NS exhibited significantly elevated scores in restricted and repetitive behaviors, social cognition, and social communication compared to TD individuals. These findings extend prior research on NS [60, 61] by highlighting variant-specific impairments across ASD-related behaviors in both NS and NSML. Furthermore, psychiatric evaluations from structured psychiatric interviews using the Kiddie Schedule for Affective Disorders and Schizophrenia indicated that children with *PTPN11*- and *SOS1*-associated NS exhibit high rates of repetitive and rigid behaviors commonly associated with ASD [14]. Interestingly, only individuals with *PTPN11*-associated NS and NSML showed significantly higher scores in social motivation (indicating lower motivation) compared to TD individuals**.** While there is limited information on behavior in NSML, previous studies did not highlight the occurrence of social difficulties in these individuals [20, 62]. In contrast, our analyses indicate that individuals with NSML appear to exhibit the most severe symptoms across ASD-related behaviors compared to TD individuals. While the various genes implicated in NS affect the RAS-MAPK pathway, *PTPN11* variants underlying NSML influence the upregulation of the PI3K-AKT-mTOR pathway [63, 64] that regulates metabolism, cell survival, and proliferation [65], and has been associated with neurodevelopmental disorders when dysregulated [66]. Together, these variant-specific differences suggest that dysregulation of the RAS-MAPK and potentially mTOR leads to distinct behavioral profiles, suggesting these pathways as potential underlying mechanisms affecting neurodevelopment.

Next, we examined the effects of genetic variants across behavioral and emotional problems from the CBCL. In line with previous research, our findings show that individuals with *PTPN11*-associated NS and NSML, as well as those with *SOS1-*associated NS variants, exhibit significantly elevated somatic complaints and thought problems compared to TD individuals [21, 67]. While the CBCL somatic complaints scale is designed to reflect subjective symptoms beyond physical illness, it is possible that the physical manifestations of NS may contribute to these elevated scores. Furthermore, we observed that individuals with *PTPN11* variant-associated NS and NSML had significantly increased scores in anxious/depressed behaviors and attention problems compared to TD individuals. Patterns of attention problems have previously been reported in RASopathies. For example, ADHD has been reported to affect approximately 48% of youth with NS, and attention problems have been commonly observed, particularly in those with a mutated *PTPN11* variant [10, 13, 14, 68, 69]. Moreover, we found that aggressive behavior was uniquely elevated in individuals with *PTPN11*-associated NS relative to TD individuals, reflecting challenges with behavioral regulation and impulsivity [59].

Next, we compared the distinct behavioral profiles across the different genetic variants. We observed notable differences in ASD-related behaviors, with the larger impairments observed in individuals with *PTPN11*-associated NS and NSML, whereas those with *RAF1-*associated NS variants were comparatively the least affected. While prior research has also reported elevated social and behavioral difficulties in individuals with *PTPN11-*associated NS [20, 21, 70], earlier studies found that individuals with *SOS1* variants did not exhibit elevated symptoms in social communication or adaptive behavior compared to those with *PTPN11*-associated NS [20, 71]. These studies also reported no significant differences in social communication or adaptive behavior between individuals with *RAF1*-associated NS variants compared to those with *PTPN11*- and *SOS1*-associated NS [20, 71]. In contrast, our exploratory results diverge from these findings, providing initial evidence of distinct variant-specific behavioral profiles. Specifically, individuals with *RAF1*-associated NS variants demonstrated significantly lower levels of ASD-related behaviors, specifically social motivation, potentially suggesting a reduced impact on social functioning. We also found that individuals with *PTPN11*-associated NSML exhibited significantly elevated scores in attention problems compared to those with *SOS1* variants, further supporting differential behavioral profiles across genetic subgroups. These discrepancies may reflect our overall larger sample size, which enabled variant-level comparisons without aggregating across variant groups, despite smaller subgroup sizes. Additionally, differences in behavioral assessment tools and measures may have contributed to these diverging findings. For example, prior studies used the Social Communication Questionnaire-Lifetime, which relies on binary “yes”/”no” responses; our study employed the SRS-2, which provides a dimensional assessment and quantifies the severity of ASD-related behaviors across specific domains. Moreover, the inclusion of the CBCL in our analyses enabled a broader evaluation of behavioral and emotional functioning, capturing a more nuanced examination of behavioral differences across variants.

Our findings provide initial evidence of variability in social and behavioral impairments across NS and NSML, suggesting specific genetic contributions to this heterogeneity. A possible explanation for this could be differences in the RAS signaling effects of associated genes or their variants on gliogenesis. Previous studies using mouse models and NS-derived induced pluripotent stem cells suggest that *PTPN11* variants may disrupt gliogenesis, either by impairing glial cell production [35, 72] or by promoting precocious gliogenesis [73]. In contrast, mouse models of *RAF1* variants show increased glial cell numbers and intact sociability [74], raising the possibility that enhanced gliogenesis may buffer against social and behavioral impairments. These findings from animal models suggest a potential mechanism that may explain the observed variability in social and behavioral impairments observed in the NS and NSML subgroups. Future research is needed to test this hypothesis and clarify the influence of gliogenesis on neurodevelopmental and behavioral outcomes in NS and NSML.

Stepping beyond previous studies observing average IQs and relatively mild cognitive delays in specific *PTPN11* variants, such as N308D and N308S [11, 15, 38, 39], we observed that behavioral functioning is influenced by SHP2 activity within our *PTPN11*-associated NS variant group. Specifically, we observed that individuals with higher levels of SHP2 fold activation (*i.e.*, higher stimulated: basal catalytic activity ratio) exhibited greater odds of clinically elevated restricted and repetitive behaviors. These findings underscore the complexity of behavioral phenotypes associated with NS, particularly in individuals with mutated *PTPN11* variants, and point to distinct mechanisms underlying genotype–phenotype relationships. Moreover, they provide preliminary insights into the potential biological markers associated with specific behavioral profiles in *PTPN11* variants. Future studies are warranted to validate these relationships and elucidate their clinical implications.

While cardiac and cognitive heterogeneity in NS have been observed across phenotypic domains, limited studies have comprehensively investigated the differential behavioral profiles associated with specific gene variants or the unique biological factors driving these variant-specific differences. By characterizing the distinct behavioral profiles associated with specific gene variants, we gain deeper insights into the variable behavioral manifestations of RAS pathway anomalies. Specifically, individuals with *PTPN11*-associated NS and NSML have the most impairments around ASD-related behaviors, social cognition, behavioral and emotional problems, and attention problems compared to TD individuals, but also compared to individuals with variants in other NS-causing genes. To better understand when these differences emerge, future longitudinal studies should examine the timing of symptom onset to determine whether age-related associations contribute to these variant-specific profiles and patterns of functioning. Additionally, the variation in SHP2’s fold activity suggests an initial relationship between restricted and repetitive behaviors – a common symptom of ASD – among individuals with *PTPN11* variants. These findings align with research in other RASopathies, such as neurofibromatosis type 1 (NF1), which shows high rates of autistic traits and attention problems, with prevalence estimates ranging from 20–30% [28, 46, 75]. Similar to NS, individuals with NF1 exhibit elevated SRS scores, ADHD symptoms, and strong familial aggregation of autistic traits, with a subset showing severe social impairments [46, 60, 75]. They also demonstrate greater symptomatology on measures of internalizing behaviors and ADHD symptoms [70], indicating broader difficulties in behavioral and emotional functioning. These patterns suggest shared neurodevelopmental mechanisms related to RAS pathway dysregulation and underscore the need for early identification and intervention across RASopathies.

Overall, this comprehensive approach offers a clearer, more nuanced understanding of how distinct gene variants influence behavioral phenotypes within NS and NSML and how biological mechanisms specific to *PTPN11-*associated NS underlie behavioral heterogeneity. While further work is needed to inform clinical applications, previous studies indicate the value of targeted therapies for genetic conditions [76]. Dietary therapies, drugs and medications, and molecular therapies are among the treatments that have been developed to target specific genetic conditions. For neurodevelopmental disorders, understanding of genotypes with more severe impairments can prompt early behavioral or pharmacological interventions, as has been observed in conditions like Fragile X Syndrome [77, 78]. Although such advancements are not yet ready for NS/NSML, this work contributes to the field by identifying the specific genotypes that are more significantly affected, representing an important first step towards intervention and treatment development.

### Limitations

There are limitations to our present study. Most importantly, our sample sizes were small, particularly for the rarest genetic variants. Given the rarity of these groups, it is difficult to recruit large samples. Global efforts across lifespan studies may also aid in increasing sample sizes of these groups. Future studies should aim to include larger numbers of participants with these rarer genetic variants causing NS or NSML to increase statistical power and enable more reliable subgroup analyses. Efforts towards open-science frameworks may also aid in increasing sample sizes of these groups in the future.

Our group sizes were unequal across the studied subgroups; most of the NS group was composed of individuals with *PTPN11* variants, the most commonly mutated gene in this disorder. Although there were unbalanced sample sizes, we employed Dunn testing, allowing us to examine subgroups with five or more individuals. Additionally, most individuals with NSML were female, while those with *RAF1* variants were male, which may have implications for behavioral outcomes. Future studies should include both sexes to enhance generalizability.

Additionally, individuals with NS or NSML with prior documented FSIQ scores below 70 were excluded due to cognitive assessment demands, limiting representativeness for those with more significant cognitive impairments. Although the IQ scores shown in our sample appear similar to prior works [18], it is possible that our participants may demonstrate IQs that are above average for the population, and this may be exacerbated given our exclusion criteria. Our NS/NSML cohort was recruited from across the United States and Canada to improve representation, though our TD group was from the local area only and tended to show higher IQ than the general population. This should be taken into consideration when viewing the findings. Inclusion of participants with a wider cognitive range would aid in producing a more representative sample.

Another limitation is the use of caregiver-report screening measures (e.g., SRS-2) without formal clinical ASD diagnoses, which may provide a broad but less detailed picture of autism-related behaviors in this sample. It should be noted that individuals with RASopathies often exhibit subthreshold autism traits that do not meet the full criteria for an ASD diagnosis [27]. Studying the presentation of these ASD-related traits, even in the absence of an ASD diagnosis, is essential for understanding their unique social challenges and informing effective interventions in the future. However, future studies would benefit from incorporating gold-standard assessments such as the Autism Diagnostic Interview-Revised (ADI-R) or the Autism Diagnostic Observation Schedule, Second Edition (ADOS-2). Inclusion of these diagnostic assessments would provide a more comprehensive and reliable evaluation of autistic trait presentation [27]. The SRS-2 was chosen for this study based on recommendations for a similar RASopathy (NF1) [27] and its application in various RASopathy studies [27]. Within the SRS-2, the total score has the strongest psychometric support, while the other subscales have less empirical validation [79]. The subscale scores were included to aid in understanding the factors contributing to overall social responsiveness in this population. Scores on the subscales should be viewed cautiously in light of this limitation.

Finally, while we explored potential biological mechanisms underlying behavioral differences (*i.e.,* SHP2 mutants’ biochemical properties), future studies should investigate these patterns across distinct gene variants to provide further insight into the genotype–phenotype relationships. Given the complexity of these syndromes, it will also be important for future research to disentangle the interplay between somatic and psychological functioning in RASopathies, to better parse out their individual contributions to observed behavioral profiles.

## Conclusions

This study underscores the importance of considering variant-specific profiling in behavioral analysis in NS and NSML. In all the different NS and NSML subgroups except *RAF1*-associated NS, significant differences in social responsiveness were observed compared to TD individuals, underscoring the severity of ASD-related behaviors in *PTPN11-*associated NS and NSML, and *SOS1*-associated NS*.* Similar patterns were observed across behavioral and emotional problems—including somatic complaints, attention problems, and thought problems—in individuals with *PTPN11*-associated NS and NSML, with the NS subgroup showing significantly greater aggressive behavior compared to TD individuals. Moreover, the observed severe impairments in social responsiveness and repetitive behaviors in individuals with *PTPN11*-associated NS suggest that the extent of SHP2 activation contributes to phenotypic variability and may inform personalized treatment strategies. Our findings extend the understanding of the *PTPN11*-associated NS genotype–phenotype relationship and phenotypic heterogeneity, though further functional studies are necessary to elucidate the impact of these variants on protein function comprehensively.

## Supplementary Information

Supplementary Materials 1

## Data Availability

The data supporting this study's findings are available from the corresponding author upon reasonable request.

## Abbreviations

ADHD: Attention deficit hyperactivity disorder
ASD: Autism spectrum disorder
CBCL: Child behavior checklist
FSIQ: Full-scale intelligence quotient
NS: Noonan syndrome
NSML: Noonan syndrome with multiple lentigines
PIQ: Performance intelligence quotient
RAS-MAPK: RAS-mitogen-activated protein kinase
SRS-2: Social responsiveness scale second edition
TD: Typically developing
VIQ: Verbal intelligence quotient
