# Supplementary material for: Genotype–phenotype correlations with autism spectrum disorder-related traits in Noonan syndrome and Noonan syndrome with multiple lentigines: a cross-sectional study
Source: Mol Autism. 2025 Oct 11;16:51. doi: 10.1186/s13229-025-00681-1 (PMC12514806; doi:10.1186/s13229-025-00681-1)
Supplement: Supplementary file 1 — Supplementary Materials 1 [file 13229_2025_681_MOESM1_ESM.docx]

**Supplementary Materials**

**Methods**

**Statistical Analysis**

***Behavioral Profiles***

To assess potential sex differences, we first evaluated the normality of behavioral outcome data across sex groups within each diagnostic group using the Shapiro-Wilk test. As distributions were non-normal, we employed the Mann–Whitney U test, a nonparametric alternative to the t-test that does not assume normality. We conducted separate Mann–Whitney tests within each diagnostic group: TD individuals, individuals with *PTPN11*-associated NS, and individuals with *SOS1*, limited to subgroups with at least five individuals per sex. Analyses were conducted across all SRS-2 subscales (measuring ASD-related traits) and CBCL subscales (measuring behavioral and emotional problems). To control for multiple comparisons, false discovery rate (FDR) correction using the Benjamini-Hochberg procedure was applied to the resulting p-values.

To assess whether differences in IQ influenced behavioral scores, we conducted an additional analysis using all SRS-2 and CBCL scores residualized for full-scale IQ (FSIQ). This analysis was limited to participants with complete FSIQ-2 scale data (NS/NSML = 108 [*PTPN11*-associated NS=74, *PTPN11*-associated NSML=7, *SOS1*-NS=12, *RAF1*-NS=6], TD = 41). We performed Kruskal-Wallis tests on the residualized SRS-2 and CBCL subscale scores to evaluate overall group differences. Where significant effects were found (*p*<.05), Dunn’s tests were used for post-hoc pairwise comparisons, with *p*-values adjusted for multiple comparisons using the Benjamini-Hochberg FDR correction within each behavioral domain. Additionally, we report Cliff’s Delta as a non-parametric measure of effect size to contextualize the magnitude of clinical significance of group differences.

**Results**

**Cognitive Functioning Across NS and NSML Variant Subgroups and TD Individuals**

We examined the distribution of cognitive scores across FSIQ, VIQ, and PIQ for TD individuals and participants with genetic variants causing NS and NSML (Table S1). For all IQ scales, most participants scored within the average range. However, as expected, a greater proportion of TD participants scored in the ‘above average’ range compared to the genetic variant groups.

**Table S1: IQ Score Distribution by Diagnosis in Individuals with NS, NSML, and Typically Developing Controls**

|  | **TD**  **(N=71)** | ***PTPN11*-NS (N=88)** | ***PTPN11-*NSML**  **(N=7)** | ***SOS1-*NS**  **(N=18)** | ***RAF1-*NS**  **(N=6)** |
| --- | --- | --- | --- | --- | --- |
| **FSIQ-2** |  | | | | |
| **2+ SD Above the Mean (≥130)** | 4 (9.8%) | 0 (0%) | 0 (0%) | 0 (0%) | 1 (16.7%) |
| **1–2 SD Above the Mean (115–129)** | 16 (39%) | 7 (9.5%) | 1 (14.3%) | 2 (16.7%) | 0 (0%) |
| **Within 1 SD of the Mean (86–114)** | 21 (51.2%) | 51 (68.9%) | 6 (85.7%) | 9 (75%) | 4 (66.7%) |
| **1–2 SD Below the Mean (71–85)** | 0 (0%) | 15 (20.3%) | 0 (0%) | 1 (8.3%) | 1 (16.7%) |
| **2+ SD Below the Mean (≤70)** | 0 (0%) | 1 (1.4%) | 0 (0%) | 0 (0%) | 0 (0%) |
| **VIQ** |  |  |  |  |  |
| **2+ SD Above the Mean (≥130)** | 6 (8.5%) | 0 (0%) | 0 (0%) | 0 (0%) | 1 (16.7%) |
| **1–2 SD Above the Mean (115–129)** | 24 (33.8%) | 3 (3.5%) | 0 (0%) | 2 (11.1%) | 0 (0%) |
| **Within 1 SD of the Mean (86–114)** | 41 (57.7%) | 67 (77.9%) | 7 (100%) | 16 (88.9%) | 4 (66.7%) |
| **1–2 SD Below the Mean (71–85)** | 0 (0%) | 15 (17.4%) | 0 (0%) | 0 (0%) | 1 (16.7%) |
| **2+ SD Below the Mean (≤70)** | 0 (0%) | 1 (1.2%) | 0 (0%) | 0 (0%) | 0 (0%) |
| **PIQ** |  |  |  |  |  |
| **2+ SD Above the Mean (≥130)** | 5 (7%) | 0 (0%) | 0 (0%) | 0 (0%) | 1 (16.7%) |
| **1–2 SD Above the Mean (115–129)** | 27 (38%) | 0 (0%) | 1 (20%) | 1 (5.6%) | 0 (0%) |
| **Within 1 SD of the Mean (86–114)** | 37 (52.1%) | 44 (71%) | 4 (80%) | 15 (83.3%) | 4 (66.7%) |
| **1–2 SD Below the Mean (71–85)** | 2 (2.8%) | 16 (25.8%) | 0 (0%) | 2 (11.1%) | 1 (16.7%) |
| **2+ SD Below the Mean (≤70)** | 0 (0%) | 2 (3.2%) | 0 (0%) | 0 (0%) | 0 (0%) |

NS, Noonan syndrome; NSML, Noonan syndrome with multiple lentigines; TD, typically developing individuals; FSIQ, Full-Scale IQ 2-scale; VIQ, Verbal IQ; PIQ, Performance IQ; SD, Standard Deviation.

The table reports the number and percentage of individuals within each IQ category for each genetic variant associated with NS and NSML, as well as for TD individuals. The values are presented as N (%) for each category.

**Sex Differences in ASD-Related Traits and Behavioral Problems Within *PTPN11*-Associated NS, *SOS1*, and TD Individuals**

To investigate potential sex differences, Mann-Whitney U tests of TD, *PTPN11*-associated NS, and *SOS1* groups revealed no significant sex differences on SRS-2 or CBCL measures (Table S2). The withdrawn/depressed subscale was slightly elevated in TD males (M = 57.29, SD = 6.42) compared to TD females (M=53.55, SD = 3.65), (*p*_uncorrected_=.013, *p*_FDR_=.471), and thought problems were slightly elevated in *SOS1* males (M=66.75, SD = 4.78) compared to *SOS1* females (M=58.17, SD = 7.43) (*p*_uncorrected_=.031, *p*_FDR_=.556).

**Table S2: Sex Differences: Mann-Whitney U Test Results Within Variant Groups on SRS-2 and CBCL Measures**

|  | **TD Female vs. TD Male** | | ***PTPN11*-NS Female vs. *PTPN11*-NS Male** | | ***SOS1* Female vs. *SOS1* Male** | |
| --- | --- | --- | --- | --- | --- | --- |
|  | *p*_uncorrected_ | *p*_FDR_ | *p*_uncorrected_ | *p*_FDR_ | *p*_uncorrected_ | *p*_FDR_ |
| **SRS-2 Measures** |  | | | | | |
| Social Responsiveness Total | 0.836 | 1.000 | 0.229 | 1.000 | 1.000 | 1.000 |
| Restricted and Repetitive Behaviors | 0.840 | 1.000 | 0.373 | 1.000 | 0.673 | 1.000 |
| Social Cognition | 1.000 | 1.000 | 0.310 | 1.000 | 0.851 | 1.000 |
| Social Communication | 0.804 | 1.000 | 0.181 | 0.932 | 0.925 | 1.000 |
| Social Motivation | 0.449 | 1.000 | 0.587 | 1.000 | 1.000 | 1.000 |
| **CBCL Measures** |  | | | | | |
| Anxious/Depressed | 0.162 | 0.932 | 0.917 | 1.000 | 0.638 | 1.000 |
| Withdrawn/Depressed | **0.013** | 0.471 | 0.857 | 1.000 | 0.161 | 0.932 |
| Somatic Complaints | 0.743 | 1.000 | 0.900 | 1.000 | 1.000 | 1.000 |
| Thought Problems | 0.749 | 1.000 | 0.300 | 1.000 | **0.031** | 0.556 |
| Attention Problems | 0.899 | 1.000 | 0.523 | 1.000 | 0.673 | 1.000 |
| Rule-Breaking Behavior | 0.592 | 1.000 | 0.138 | 0.932 | 0.540 | 1.000 |
| Aggressive Behavior | 0.695 | 1.000 | 0.067 | 0.809 | 0.963 | 1.000 |

NS, Noonan syndrome; TD, typically developing individuals; SRS-2, Social Responsiveness Scale; CBCL, Child Behavior Checklist

Bolded values represent significant *p*-values (*p* < .05).

**Variant-Specific Differences in ASD-Related Traits and Behavioral-Emotional Problems Compared to TD Individuals, Controlling for IQ**

To determine whether behavioral differences persisted after accounting for cognitive ability, we residualized SRS-2 and CBCL scores on full-scale IQ (FSIQ; Table S3). Kruskal-Wallis tests revealed significant group differences across all SRS-2 subscales except for social motivation. For CBCL subscales, significant group differences were observed in somatic complaints, thought problems, and attention problems. Post hoc Dunn’s tests indicated that the group differences were like our earlier findings, where we did not control for IQ (Table 4). Individuals with *PTPN11*-associated NS, *PTPN11*-associated NSML, and *SOS1* variants continued to show significantly higher scores on several SRS-2 subscales compared to TD individuals (Table S3). However, unlike our earlier analysis, differences in restricted and repetitive behaviors for the *SOS1* variant subgroup and social cognition for *PTPN11*-associated NS were no longer statistically significant after controlling for FSIQ. Across CBCL measures, post hoc Dunn’s tests demonstrated that group differences in behavioral and emotional problems persisted for individuals with *PTPN11*-associated NSML and *SOS1* variants, while effects for *PTPN11*-associated NS were attenuated after controlling for FSIQ.

These findings suggest that adjusting for IQ attenuated some group differences; however, many distinct differences between the groups remained, indicating that these social and behavioral differences are not attributable to cognitive ability. Furthermore, the absence of FSIQ data for all participants limited the sample sizes in our FSIQ-controlled analysis, potentially masking some of the effects.

**Table S3: FSIQ-Residualized Kruskal-Wallis and Dunn’s Test Results Comparing Genetic Variants to Typically Developing Individuals**

|  | **Kruskal-Wallis** | ***PTPN11-*NS vs. TD** | | ***PTPN11-*NSML vs. TD** | | ***SOS1*-NS vs. TD** | | ***RAF1*-NS vs. TD** | |
| --- | --- | --- | --- | --- | --- | --- | --- | --- | --- |
|  | ***P-value*** | ***P_FDR_*-value** | **Effect Size** | ***P_FDR_*-value** | **Effect Size** | ***P_FDR_*-value** | **Effect Size** | ***P_FDR_*-value** | **Effect Size** |
| **SRS-2 Measures** |  |  | | | |  | | | |
| Social Responsiveness Total | **0.003** | **0.018** | 0.305 | **0.007** | 0.796 | **0.044** | 0.329 | 0.317 | -0.267 |
| Restricted and Repetitive Behaviors | **0.001** | **0.004** | 0.393 | **0.004** | 0.800 | 0.060 | 0.333 | 0.422 | -0.233 |
| Social Cognition | **0.008** | 0.056 | 0.234 | **0.009** | 0.804 | 0.079 | 0.300 | 0.285 | -0.275 |
| Social Communication | **0.008** | **0.034** | 0.284 | **0.028** | 0.707 | **0.032** | 0.425 | 0.370 | -0.175 |
| Social Motivation | 0.090 | Not Sig. | 0.159 | Not Sig. | 0.593 | Not Sig. | 0.127 | Not Sig. | -0.175 |
| **CBCL Measures** |  |  | | | | | | | |
| Anxious/Depressed | 0.094 | Not Sig. | 0.046 | Not Sig. | 0.700 | Not Sig. | 0.000 | Not Sig. | -0.325 |
| Withdrawn/Depressed | 0.868 | Not Sig. | 0.026 | Not Sig. | 0.161 | Not Sig. | -0.064 | Not Sig. | -0.218 |
| Somatic Complaints | **0.013** | 0.086 | 0.198 | **0.005** | 0.670 | **0.016** | 0.466 | 0.249 | 0.299 |
| Thought Problems | **0.042** | 0.334 | 0.111 | **0.001** | 0.722 | 0.108 | 0.312 | 0.525 | -0.171 |
| Attention Problems | **0.041** | 0.085 | 0.198 | **0.005** | 0.670 | 0.128 | 0.295 | 0.726 | -0.094 |
| Rule-Breaking Behavior | 0.370 | Not Sig. | -0.229 | Not Sig. | -0.245 | Not Sig. | -0.165 | Not Sig. | -0.282 |
| Aggressive Behavior | 0.618 | Not Sig. | -0.021 | Not Sig. | 0.311 | Not Sig. | 0.034 | Not Sig. | -0.205 |

NS, Noonan syndrome; NSML, Noonan syndrome with multiple lentigines; TD, typically developing individuals; SRS-2, Social Responsiveness Scale Second Edition.

Bolded values represent significant *p*-values (*p* < .05). Not Sig. (not significant) is presented in cases where Kruskal-Wallis *p*>.05, and post-hoc Dunn’s tests were therefore not performed. Kruskal-Wallis *p*-values are uncorrected. Pairwise Dunn’s tests *p*-values are FDR-corrected. Cliff’s delta effect sizes are reported, where a small effect size is δ = 0.147 to 0.330, a small to medium effect is δ =0.330 to 0.474, and a large effect is δ >0.474.

**Table S4: Kruskal-Wallis Uncorrected Results Comparing SRS and CBCL Scores Across Genetic Variants Only (*PTPN11*-associated NS, *PTPN11*-associated NSML, *RAF1*, *SOS1*)**

| **Measure** | ***P*-value** |
| --- | --- |
| **SRS-2 Measures** |  |
| Social Responsiveness Total | **0.035** |
| Restricted and Repetitive Behaviors | 0.081 |
| Social Cognition | **0.049** |
| Social Communication | 0.101 |
| Social Motivation | 0.071 |
| **CBCL Measures** |  |
| Anxious/Depressed | 0.086 |
| Withdrawn/Depressed | 0.219 |
| Somatic Complaints | 0.078 |
| Thought Problems | 0.130 |
| Attention Problems | **0.032** |
| Rule-Breaking Behavior | 0.525 |
| Aggressive Behavior | 0.353 |

NS, Noonan syndrome; NSML, Noonan syndrome with multiple lentigines; TD, typically developing individuals; SRS-2, Social Responsiveness Scale Second Edition; CBCL, Child Behavior Checklist.

Bolded values represent significant *p*-values (*p* < .05).

**Table S5: Information on Pathogenic Variants in *PTPN11-*associated NS and NSML Variants and TD Individuals**

| Amino Acid Substitution | Number of subjects in the cohort carrying the variant | Class of Location of Amino Acid Substitution | Basal Activity | Stimulated catalytic activity (BTAM, 10 μM) | Fold Activation |
| --- | --- | --- | --- | --- | --- |
| **WT protein** |  |  |  |  |  |
|  | 71 (TD) |  | 1 | 3.7 | 3.70 |
| ***PTPN11*-associated NS** |  |  |  |  |  |
| T42A | 2 | Class B | 1.1 | 13.8 | 12.55 |
| N58D | 2 | Class A | 2 | 10.2 | 5.10 |
| N58H | 2 | Class A | 1.1 | 10.2 | 9.27 |
| T59A | 1 | Class A | 1.3 | 8.5 | 6.54 |
| G60A | 1 | Class A | 1.4 | 10.1 | 7.21 |
| D61G | 2 | Class A | 2.8 | 9.3 | 3.32 |
| D61N | 1 | Class A | 1.5 | 12.3 | 8.20 |
| Y62D | 3 | Class A | 3 | 13 | 4.33 |
| Y63C | 11 | Class A | 2.4 | 11.1 | 4.63 |
| E69Q | 1 | Class A | 1.2 | 7.7 | 6.42 |
| K70R | 1 | Class A | 1.4 | 5.6 | 4.00 |
| A72G | 1 | Class A | 1.5 | 11.2 | 7.47 |
| T73I | 1 | Class A | 2.0 | 11.4 | 5.70 |
| E76D | 1 | Class A | 3 | 12.4 | 4.13 |
| Q79R | 2 | Class A | 1.2 | 8.2 | 6.83 |
| D106A | 3 | Class C | 1.2 | 8 | 6.67 |
| E139D | 5 | Class B | 1.2 | 10.2 | 8.50 |
| L261P | 1 | Class A | 0.9 | 5.1 | 5.67 |
| R265Q | 3 | Class A | 1.9 | 9.0 | 4.74 |
| I282M | 2 | Class A | 1.2 | 8.2 | 6.83 |
| I282V | 3 | Class A | 2.8 | 19 | 6.79 |
| F285S | 1 | Class A | 0.7 | 6.8 | 9.71 |
| N308D | 16 | Class A | 1.3 | 10.1 | 7.77 |
| N308S | 10 | Class A | 0.7 | 5.3 | 5.57 |
| P491L | 3 | Class A | 1.1 | 9.7 | 8.82 |
| S502T | 1 | Class A | 1.8 | 7.1 | 3.94 |
| G503R | 3 | Class A | 7.9 | 9.5 | 1.20 |
| M504V | 5 | Class A | 1.4 | 7.8 | 5.57 |
| ***PTPN11-*associated NSML** |  |  |  |  |  |
| Y279C | 1 | Class D | 0.2 | 0.6 | -* |
| G464A | 1 | Class D | 0.05 | 0.2 | -* |
| T468M | 5 | Class D | 0.1 | 0.2 | -* |

WT, Wild Type; TD, typically developing individuals; NS, Noonan Syndrome; NSML, NSML, Noonan syndrome with multiple lentigines.

Phosphatase assay was performed to functionally characterize pathogenic *PTPN11*-associated NS and NSML variants. Catalytic activity was measured as pmoles of phosphate released using pNPP as a substrate. Fold activation for each pathogenic variant in individuals with *PTPN11* variants is determined by dividing the stimulated phosphate release activity by the basal unstimulated activity.

*, not relevant in the context of *PTPN11*-associated NSML.

Class A (N=78) includes residues involved in the N-SH2/PTP interaction (within the N-SH2 and PTP domain); Class B (N=7) includes residues implicated in binding to partners (Thr42 in the N-SH2 domain and Glu139 in the C-SH2 domain); Class C (N=3) includes residues located in the linker connecting the two SH2 domain (Asp106); Class D (N=7) includes residues mutated in *PTPN11*-associatd NSML (Tyr279, Gly464, Thr468).

**Table S6: The Logistic Regression Results for the SRS-2 and CBCL Measures for Basal Unstimulated Activity *PTPN11* variant-associated NS**

|  | **Basal Unstimulated Activity** | | |
| --- | --- | --- | --- |
|  | **Odds Ratio** | ***P*_uncorrected_** | ***P*_FDR-corrected_** |
| **SRS-2 Measures** |  | | |
| Social Responsiveness Total | 0.81[0.31-1.36] | 0.571 | 0.571 |
| Restricted and Repetitive Behaviors | 0.60[0.22-1.12] | 0.233 | 0.485 |
| Social Cognition | 0.58[0.14-1.27] | 0.360 | 0.485 |
| Social Communication | 1.19[0.76-1.71] | 0.364 | 0.485 |
| **CBCL Measures** |  | | |
| Anxious/Depressed | 1.84[1.16-3.01] | **0.007** | 0.051 |
| Withdrawn/Depressed | 0.31[0.01-1.68] | 0.456 | 0.798 |
| Somatic Complaints | 0.24[0.00-1.64] | 0.402 | 0.798 |
| Thought Problems | 0.91[0.22-1.66] | 0.845 | 0.845 |
| Attention Problems | 1.10[0.47-1.73] | 0.752 | 0.845 |
| Rule-Breaking Behavior | 0.32[0.00-2.12] | 0.600 | 0.840 |
| Aggressive Behavior | 1.25[0.54-2.03] | 0.430 | 0.798 |

SRS-2, Social Responsiveness Scale Second Edition; CBCL, Childhood Behavior Checklist.

Bolded values represent significant *p*-values (*p*<.05).
